# An archaeal sRNA targeting *cis*- and *trans*-encoded mRNAs via two distinct domains

**DOI:** 10.1093/nar/gks847

**Published:** 2012-09-08

**Authors:** Dominik Jäger, Sandy R. Pernitzsch, Andreas S. Richter, Rolf Backofen, Cynthia M. Sharma, Ruth A. Schmitz

**Affiliations:** ^1^Institut für Allgemeine Mikrobiologie, Christian-Albrechts-Universität zu Kiel, Am Botanischen Garten 1-9, 24118 Kiel, ^2^Zentrum für Infektionsforschung, Universität Würzburg, Josef Schneider-Str. 2/Bau D15, 97080 Würzburg and ^3^Institut für Informatik, Albert-Ludwigs-Universität Freiburg, Georges-Koehler-Allee 106, 79110 Freiburg, Germany

## Abstract

We report on the characterization and target analysis of the small (s)RNA_162_ in the methanoarchaeon *Methanosarcina mazei*. Using a combination of genetic approaches, transcriptome analysis and computational predictions, the bicistronic MM2441-MM2440 mRNA encoding the transcription factor MM2441 and a protein of unknown function was identified as a potential target of this sRNA, which due to processing accumulates as three stabile 5′ fragments in late exponential growth. Mobility shift assays using various mutants verified that the non-structured single-stranded linker region of sRNA_162_ (SLR) base-pairs with the MM2440-MM2441 mRNA internally, thereby masking the predicted ribosome binding site of MM2441. This most likely leads to translational repression of the second cistron resulting in dis-coordinated operon expression. Analysis of mutant RNAs *in vivo* confirmed that the SLR of sRNA_162_ is crucial for target interactions. Furthermore, our results indicate that sRNA_162_-controlled MM2441 is involved in regulating the metabolic switch between the carbon sources methanol and methylamine. Moreover, biochemical studies demonstrated that the 5′ end of sRNA_162_ targets the 5′-untranslated region of the *cis*-encoded MM2442 mRNA. Overall, this first study of archaeal sRNA/mRNA-target interactions unraveled that sRNA_162_ acts as an antisense (as)RNA on *cis*- and *trans*-encoded mRNAs via two distinct domains, indicating that *cis*-encoded asRNAs can have larger target regulons than previously anticipated.

## INTRODUCTION

In recent years, an increasing number of non-coding RNAs have been shown to participate in various regulatory cellular processes in both pro- and eukaryotes, mainly acting as post-transcriptional ribo-regulators. Although the abundant class of eukaryotic miRNAs mainly act by base-pairing to the 3′- untranslated region (UTR) or coding sequence (CDS) of the cognate target-mRNA, most of the functionally characterized bacterial small regulatory RNAs (sRNAs) target the 5′-UTR of mRNAs (1–3). Besides several sRNAs which directly modulate protein activity, the majority of functionally characterized sRNAs from bacteria belong to the class of *trans*-encoded antisense RNAs. In most cases, they bind with only partial sequence complementarities to the 5′-UTR of their target genes, and lead to translational repression by masking the ribosome binding site (RBS). Consequently, the association of the ribosomal 30S subunit to the RBS is inhibited, which is often coupled to target destabilization by RNases (2). However, repressive translational control and induction of mRNA degradation is not limited to direct base-pairing with the RBS, because some sRNA have been shown to target sequences that are located far upstream of the RBS, within the CDS or in the intergenic region of polycistronic mRNAs (4–9). Besides repression of target genes, bacterial sRNAs have also been demonstrated to up-regulate gene expression by disruption of inhibitory secondary structures which sequester the RBS, known as anti-antisense mechanism (10–12). The underlying sRNA-target interactions together with the coupled sRNA decay are often facilitated by the bacterial RNA chaperone Hfq, which contains a Sm-like domain (13–15). Additionally, bacterial sRNAs have been shown to target multiple, often functionally related targets, some of which are regulators themselves, and a single sRNA can encompass the expression of large regulons (reviewed in 16,17).

Although first examples of so-called *cis*-encoded antisense (as)RNAs, which are encoded on the opposite strand of their target genes, were studied already in the early 1980s on plasmids and transposons (18–20), asRNAs are less well characterized in comparison with their *trans*-acting counterparts. Such asRNAs generally act on their targets independently of Hfq, possibly due to the high extent of base-pairing between the sRNA and their target mRNAs. They overlap with their targets either at the 5′-UTR or 3′-UTR or within the center of the target mRNA. For asRNAs diverse regulatory mechanisms have been described, e.g. alteration of target stability, interplay with RNases, or interference with transcription (for details see 21 and 22). The massive detection of antisense transcription in various genome-wide transcriptome studies clearly suggests a more fundamental role in prokaryotic cells; e.g. in *Helicobacter pylori* for 46% of all ORFs antisense transcription was detected (23). Although a massive antisense transcription was also demonstrated in other human pathogens such as *Listeria monocytogenis* or *Staphylococcus aureus*, and also in the cyanobacterium *Synechocystis sp.* PCC 6803, the total impact of asRNA regulation is still not yet understood (24–27).

In contrast to bacteria until recently no sRNAs had been identified in archaea, disregarding the role of the extensively studied eukaryotic like small nuclear RNAs (snoRNAs), which participate in ribosomal RNA biogenesis and tRNA maturation (28–31). Currently, several archaea have been examined for the presence of sRNAs on a genome-wide scale either by RNomics approaches or using high throughput sequencing (HTPS) techniques, e.g. RNA-Seq approaches (HTPS of cDNAs), resulting in the detection of high numbers of sRNA candidates (32–35). To study the impact of sRNAs and to get a deeper insight into transcriptional and post-transcriptional regulation in the methanogenic archaeon *Methanosarcina mazei* Gö1, specifically in response to nitrogen (36), we have recently applied a newly developed differential RNA-Seq (dRNA-Seq) approach for the analysis of primary transcriptomes (23). This approach resulted in the identification 248 sRNA candidates, a high number of which were confirmed by northern blot analysis (36). Here, we report on the functional characterization and target analysis of the abundant sRNA_162_. Using genetic, biochemical and computational approaches, we demonstrate that sRNA_162_ interacts with both, a *cis*- and a *trans*-encoded target mRNAs via two distinct domains, a mechanism which has not been shown for any studied sRNA in prokaryotes so far.

## MATERIALS AND METHODS

### Strains and plasmids

Strains and plasmids, which were used in this study, are listed in Supplementary Table S1. Plasmid DNA was transformed *M. mazei* as described by Ehlers *et al.* (37).

### Construction of *M. mazei* mutants and generation of plasmids

pRS699 was constructed by polymerase chain reaction (PCR) amplification of sRNA_162_ including its native promoter and terminator from genomic *M. mazei* DNA using primers s162-XhoI.for and s162-KpnI.rev (Supplementary Table S2). The 628 bp PCR-fragment was TA cloned into the pCR4-TOPO (Invitrogen Karlsruhe, Germany), resulting in pRS699. After restriction with XhoI and KpnI the fragment was inserted into the multiple cloning site of pWM321 (38). The resulting plasmid (pRS474) and mutant derivatives (discussed later) were transformed into *M. mazei* by liposome-mediated transformation as previously described (37,39). Puromycin-resistant transformants were selected as colonies that grew on minimal medium plates with trimethylamine as the carbon and energy source plus 5 µg puromycin/ml during incubation.

Using pRS699 as template the sRNA_162_ M1 and M2 mutants were generated by site-directed mutagenesis with the primer sets s162-Mut1-for/rev or s162-Mut2-for/rev resulting in pRS702 and pRS704. Further mutant derivatives were constructed by inverse PCR using pRS699 as PCR template: Replacement of the linker region between SL2 and SL3 by poly(T) with primers s162-Mut3-for/rev (pRS706) and the deletion of the 3′ end retaining the 5′ fragment (nucleotide 1–65, pRS701) and 3′ fragment (nucleotide 65–191, pRS708) of sRNA_162_ with s162-Mut4-for/rev and s162-Mut5-for/rev, respectively. The amplified products were religated and cloned into *E. coli*. Using this cloning strategy, the cognate sRNA variants kept their native sRNA_162_ promoter and terminator sequences. By using the aforementioned XhoI and KpnI restriction sites, the mutated sRNA_162_ variants were inserted into pWM321 as described earlier (pRS701, pRS703, pRS705, pRS707, pRS709).

For the construction of the chromosomal sRNA_162_, deletion mutant by homologous recombination in *M. mazei*, plasmid pRS650 was constructed as follows. The flanking upstream region (∼1 kbp) of sRNA_162_ was amplified by PCR using s162 Del1 XhoI and s162 Del2 EcoRI. The flanking downstream region (∼1 kbp) was amplified by PCR using s162 Del3 EcoRI and s162 Del4 XbaI. Both PCR products were restricted with the indicated restriction enzymes and separately ligated to correspondingly linearized pBluescript SK+ resulting in pRS648 and pRS649, respectively. The ∼1.8 kbp EcoRI fragment of pRS207 carrying the pac cassette from *Methanococcus voltae* (40) conferring puromycin resistance was ligated in a three body ligation together with the upstream fragment (XhoI/EcoRI restricted) into the XhoI/EcoRI restricted plasmid pRS649 generating plasmid pRS650. pRS650 was transformed into *M. mazei* using a liposome-mediated transformation protocol as described previously (37). Southern blot analyses of genomic DNA from puromycin-resistant transformants were used to verify pac insertion as described by Ehlers *et al.* (37).

The T7-hammerhead ribozyme fusion of sRNA_162_ and sRNA_162Δ63-88 _was generated by commercial gene synthesis (Eurofins MWG Operon, Ebersberg, Germany). TA cloning of both of the products into vector pCR4-TOPO (Invitrogen Karlsruhe, Germany) yielded plasmids pRS765 and pRS766, respectively.

To fuse a T7 promoter to MM2440-41, the operon was amplified with genomic DNA as template as described (see *in vitro* transcription). The PCR product was TA cloned into pCR4-TOPO (pRS767) and the obtained plasmid was subjected to site-directed mutagenesis with primers 2441-com_Mut1for/rev (pRS768) and 2441-com_Mut2for/rev (pRS769) to obtain compensatory mutants.

### RNA isolation

Exponentially or stationary phase cultures (50–70 ml) were harvested at 4°C and RNA was isolated by phenol extraction as recently described (36) or using the RNeasy Midi Kit according to the manufacturer’s instructions (Qiagen, Hilden, Germany) and published for *M. mazei* (41,42).

### Northern blot analysis

Cells were grown under different nitrogen (N) availabilities and harvested at different growth stages (N sufficiency: early exponential phase, OD_600_ = 0.2; mid-exponential phase, OD_600_ = 0.5, stationary phase, OD_600_ = 0.7; N limitation: mid-exponential phase, OD_600_ = 0.2). Northern blot analysis followed using the recently described protocol (36). sRNA_162_ and its potential processed derivatives were detected with a 5′-end radioactive labeled ssDNA oligo probe (Supplementary Table S2) and 5S rRNA as described in Jäger *et al.* (36). Full length riboprobe preparation followed the same procedure as described for T7 *in vitro* transcription, except that the MAXIscript T7 kit (Ambion; Applied Biosystems, Foster City, CA) was used according to the manufacturer. To amplify a T7 promoter containing PCR product which serve as template for antisense probe synthesis the primer pairs asPs162 for and asPs162rev, or asPs171 for and asPs171rev, were used, respectively (Supplementary Table S2).

### Rapid amplification of cDNA ends analysis

RACE (*r*apid *a*mplification of *c*DNA *e*nds) analysis was performed to determine the transcriptional start site (TSS) as well as the transcript termination site of sRNA_162_. The 5′-RACE system (Invitrogen, Karlsruhe, Germany) was used according to manufacturer’s instructions with oligonucleotide 5′-RACE 2441 as gene-specific primer to determine the TSS of sRNA_162_ (Supplementary Table S2). 3′-RACE analysis followed the 3′-RACE System (Invitrogen, Karlsruhe, Germany) according to manufacturer’s instructions after polyadenylation of RNA with Poly(A)-polymerase (NEB, Schwalbach, Germany). The precipitated RNA was resuspended in 11 -µl DEPC-H_2_O and directly used for 3′ RACE using the oligonucleotide 3′-RACE 2441 #2 as gene-specific primer (Supplementary Table S2). PCR products were subjected to TOPO-TA cloning using the TOPO-TA Cloning Kit (Invitrogen, Karlsruhe, Germany). 5′ end and 3′ end of sRNA_162_ were determined by DNA sequencing of both strands. For 5′-end analysis of the operon MM2440-41, the FirstChoice RLM-RACE kit (Ambion; Applied Biosystems) was used, according the manufacturer’s instructions. The oligonucleotides 5′-2441-RLM (*R*NA *l*inker *m*ediated)-out and 5′-2441-RLM-in were used as gene-specific primers to determine the TSS. In the same way, the oligonucleotides 5′-2442-RLM-out and 5′-2442-RLM-in or 5′-2446-RLM-out and 5′-2446-RLM-in were used to determine the TSS of MM2442 or MM2446, respectively (Supplementary Table S2).

### *In vitro* T7 transcription, purification and 5′-end labeling of RNA

Templates for *in vitro* transcription were either amplified from genomic *M. mazei* wild-type (wt) DNA or from plasmids carrying the respective constructs (Supplementary Table S1). Primer sequences are summarized in Supplementary Table S2. Plasmids carrying sRNA_162 _(or derivatives, discussed earlier) were PCR amplified simultaneously fusing a T7 promoter to the PCR product. For the 3′ end of sRNA_162_, the primer pair T7-s162-SHORT-for and sRNA162-T7.rev was used, whereas the other T7 sRNA_162_ fusions were amplified with T7-HH-s162-for and sRNA162-T7.rev instead. Similar, T7-2440-41-for and T7-2440-41-rev1 or T7-2440-41-rev2 were used for the T7 fusions of the long and short versions of mRNA MM2440-2441, respectively. T3-MM2446-for and rev were used for the amplification and the incorporation of a T3 promoter to MM2446. *In vitro* transcription was performed using the MEGAscript T7 (or T3) kit (Ambion; Applied Biosystems) according to the manufacturer. Following extraction with phenol:chloroform:isopropanol (25:24:1 v/v) and purification with G-25 column, the RNA was precipitated overnight at −20°C with 3 vol. of ethanol and 20 µg glycogen (Applied Biosystems). RNA quality and integrity were checked on a denaturing polyacrylamide gel (6% PAA, 7 M Urea). The *in vitro* transcribed RNA was dephosphorylated with FastAP (Thermosensitive Alkaline Phosphatase, MWG Fermentas), according to the manufacturer and radioactively labeled at the 5′ end as described in Jäger *et al.* (36), additionally supplementing the labeling reaction with 40 U of RNasin (Promega, Mannheim, Germany).

### A hammerhead ribozyme transcriptional fusion with sRNA_162_

Usually, *in vitro* transcripts were created by incorporating a T7 promotor into the oligonucleotides used for PCR of the template or by cloning of the respective PCR product into a T7 promotor containing vector. To ensure efficient transcription with high yield, the PCR-based T7 fusion usually introduces 3 Gs at the TSS (+1 site). To prevent the addition of non-sequence specific nucleotides into the transcripts and avoid the introduction of artificial structural changes, we used a method recently described by Holmquist *et al.* (43), based on the work of the Theobald-Dietrich and co-workers (44). Basically, we constructed and synthesized a DNA template of sRNA_162 _for T7 polymerase, where the promotor was fused to a hammerhead ribozyme (Supplementary Figure S3), further linked to the sequence of choice, here sRNA_162_. As soon as polymerization of the transcript starts, the RNA is simultaneously folded into its hammerhead shape. When folding is completed, the ribozyme is autocatalytically self-cleaved, whereas the transcription reaction of the downstream sequence continuous. Thus, the fusion guarantees transcription at the native +1 site of sRNA_162_, and can be engineered for any template choice, thereby providing an efficient method for the preparation of native RNA transcripts (Supplementary Figure S3).

### *In vitro* structure analysis

Structure probing of sRNAs and mRNAs were conducted in total volume of 10 µl. Before RNase T1 and A cleavage the RNA (∼0.1 pmol) was denatured for 1 min at 95°C and chilled on ice for 5 min, then 10× Structure Buffer (Ambion; Applied Biosystems), 1 µg yeast RNA (Ambion; Applied Biosystems) were added. Following a renaturation step for 10 min at 37°C, 25 mM lead(II)acetate (Carl Roth, Karlsruhe), 0.01 U RNase T1 (Ambion; Applied Biosystems) or RNAse A (Ambion; Applied Biosystems) was added and incubated for to 2, 3 and 5 min. The reactions were either stopped with 0.2 M EDTA and precipitated, or by directly adding gel loading buffer II (Ambion; Applied Biosystems).

For RNase T1 ladders, the labeled RNA (∼0.2 pmol) was incubated for 1 min at 95°C in 1× Sequencing Buffer (supplied with RNase T1). Subsequently, 0.1 U RNase T1 was added to mix and further incubated for 5–10 min at 37°C. OH ladders were generated by incubating ∼0.2 pmol labeled RNA for 5 min at 95°C in 1× alkaline hydrolysis buffer (supplied with RNase T1). Reactions were stopped as described earlier. The samples were analysed on 8% polyacryl amide/7M urea sequencing gels and visualized with a phosphoimager (FLA-5000 Series, Fuji).

### *In vitro* binding assays

Electro mobility shift assays were conducted in a total volume of 10 µl in the presence of 1X structure buffer (Ambion; Applied Biosystems) and 1 µg yeast RNA (Ambion; Applied Biosystems). 20 pmol of *in vitro* transcribed RNA were dephosphorylated and radioactively 5′ labeled as described earlier. 5 nM of the labeled RNA were incubated in presence with increasing amounts of the target RNA (8, 16, 32, 125, 250, 500, 1000, 2000 nM) for 15 min at 37°C and subsequently separated on native 6–8% poly acrylamide gel in a 0.5× Tris–borate buffer system (0.45 M, pH 8.0). Gels were analysed using a phosphoimager (FLA-5000 Series, Fuji).

### Transcriptome analyses

For genome-wide expression profiling genome wide microarrays representing 97% of the ORFs were used (41,45). Total RNA was extracted from *M. mazei* sRNA_162_-overexpressing cultures and the wt containing pWM321 grown with 150 mM methanol as carbon and energy source as described in Veit *et al.* (41). In general, RNA was extracted at turbidities OD_600_ = 0.5. Purified RNA was converted to cDNA and labeled by fluorescent Cy-3 and Cy-5 dyes using the CyScribe first-strand cDNA labeling kit (GE Healthcare, Freiburg, Germany) as described (41,45). Microarray slides were incubated with aliquots of the cDNA preparations at 42°C overnight (Lucidea SlidePro hybridization chamber, GE Healthcare), for details of the hybridization and wash procedures see Hovey *et al.* (45). Signal intensities were analysed using a GenePix 4100A scanner and data normalization and evaluation was performed using the GenePix Pro software version 6.0 (Axon Instruments, Union City, USA) as described recently (41,42). RNA isolated from three independent cultures were used independently in pairs of labeling reactions and, for one pair, a dye-swap experiment was also performed. Only if a transcript had an abundance difference of at least 3-fold in the comparisons of wt RNA was the difference reported here to be significant.

Quantitative reverse transcriptase PCR (qRT-PCR) assays were performed with a QuantiTect Probe RT-PCR Kit (Qiagen, Hilden, Germany) using a 7300 real-time PCR system (ABI, Foster City, USA) and at least three independent RNA preparations for each strain as described (42,46). Primer sets used including the control genes are listed in the Supplementary Table S2. The fold change in transcript abundance for genes of interest was determined by comparison with the threshold cycle (C_t_) of transcripts of three control genes (MM1621, MM2181, MM1215). The fold change in the abundance of a transcript was calculated using the formula fold change* = *2^−ΔΔ^*^Ct^* as described (47).

### Computational target predictions

The ribosome binding site (RBS) sequence positions for all annotated *M. mazei* genes were predicted following the approach of (48). Significant RBS locations were obtained for 55.2% of the genes. Putative base pair interactions with sRNA_162_ were predicted for all ORFs with at least 3-fold change in transcript level in the microarray analysis. For ORFs organized in an operon structure, the first ORF of the respective operon was also included. Sequences used for the interaction prediction contained the full length 5'-UTR (36) and additional 100 nt of the CDS. If the exact 5'-UTR start was unknown, 200 nt upstream of the annotated translation start were used instead. Interaction predictions were computed with the tool IntaRNA (49,50) requiring an interaction seed of eight consecutive base pairs. All mRNA subsequences were folded locally in a 100 nt window with 50 nt maximal base pair span. For those ORFs which showed reduced transcript levels in the sRNA_162_ overproducing mutant using the microarray, we selected all interactions that were predicted to be located in the mRNA region from −39 in the 5'UTR to +19 in the CDS, which is the maximal region covered by ribosomes (51). For all ORFs with increased transcript levels, we studied the influence of the putative interaction on the accessibility of the predicted RBS sequence. All interactions predicted to be located upstream of the RBS sequence were included. A measure for the accessibility of a subsequence is its probability to be unpaired, i.e. free of intra-molecular base pairings. We computed the unpaired probability of the RBS sequence before and after the putative interaction of the mRNA with the sRNA_162_ (49). When the change in this unpaired probability was greater than 0.001, we assumed that the structural rearrangement in the 5'-UTR caused by the interaction increases the accessibility of the RBS to activate the translation of the gene.

## RESULTS

### Characterizing sRNA_162_

**Figure 1.**
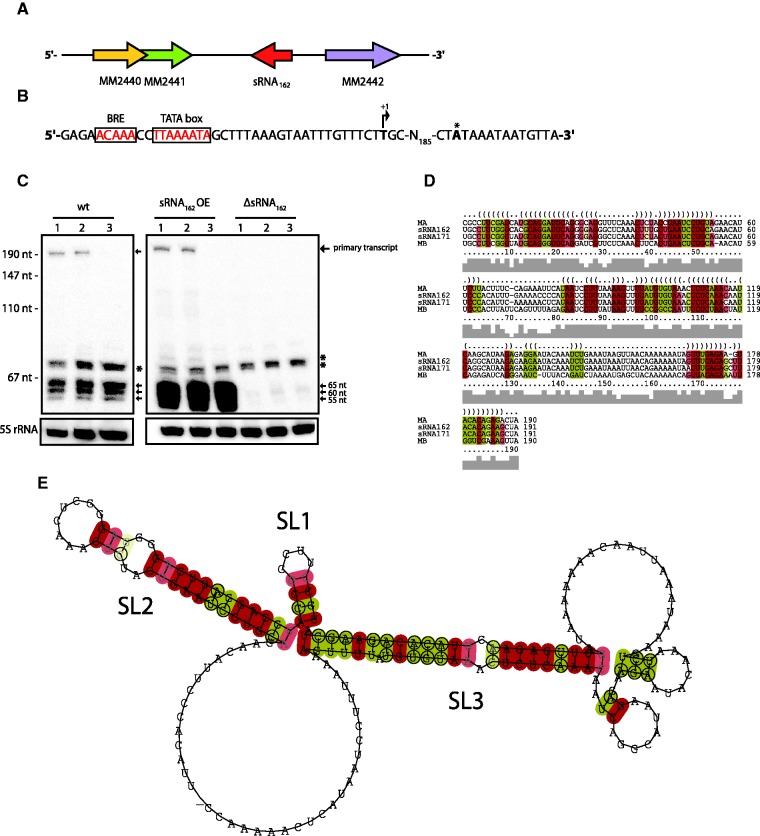
Characterization of sRNA_162_. (**A**) Genomic context of sRNA_162_. (**B**) Promotor and terminator region of sRNA_162_. The 5′ end (+1) of sRNA_162_ was determined by 5′-RLM RACE, as well as the 3′ end (*) by 3′-RACE analysis. (**C**) Northern blot analysis of total RNA of wt and sRNA_162_ mutant strains grown with methanol from early exponential phase (OD_600_ of 0.2, lanes 1), mid exponential phase (OD_600_ of 0.5, lanes 2) and stationary phase (OD_600_ of 0.7, lanes 3). The three 5′-fragments of sRNA_162_ and the primary transcript are indicated by arrows. Cross-hybridization of the sRNA_162_ probe is indicated by an asterisk. The lower panel shows the expression of 5S rRNA of the respective RNA preparation. wt, wild-type strain; sRNA_162_ OE, sRNA_162_ overexpressed from pWM321 in wt; and ΔsRNA_162_, chromosomal sRNA_162_ deletion mutant. (**D**) Secondary structure alignment of sRNA_162_ homologues identified by computer-based searches of related *Methanosarcina* species performed with LocARNA (52), Ma, sRNA_162_ homologue of *M. acetivorans* C2A; Mb, *M. barkeri* strain fusaro. (**E**) Consensus secondary structure prediction by RNalifold (53). SL, stem loop.

We have recently identified sRNA_162_ in our dRNA-seq approach to study the transcriptome of *M. mazei* (36). Because northern blot analysis revealed growth phase-dependent expression and processing of this particular sRNA, we selected sRNA_162_ for further functional characterization and analysis of its target genes. The sRNA_162_ gene is located in the **739 **bp intergenic region between MM2441, encoding a transcriptional regulator of the ArsR family, and MM2442, encoding a conserved protein of unknown function (Figure 1A). The TSS and termination site of sRNA_162 _were determined by 5′ RLM and 3′ RACE, respectively (see ‘Materials and Methods’ section), and revealed a primary transcript of 191 nucleotides (nt) (Figure 1B and C). In addition, the RACE approach revealed the presence of the homologous sRNA_171_ with exactly the same transcript length, which is encoded in the intergenic region between MM2448 and MM2449 (discussed later). Expression of both RNAs was confirmed by northern blot analysis, and further revealed that the constitutively transcribed full length sRNA_162_ is processed into three different stable 5′ fragments (nt 1–65, 1–60, 1–55). In stationary growth phase, complete processing of sRNA_162_ is observed independently of the supplied carbon source (Figure 1C; Supplementary Figure S1A). This is possibly achieved by exonucleases which degrade the 3′ fragment, since the corresponding, processed 3′ fragment(s) are neither detectable by northern blot analysis nor qRT-PCR (data not shown). In addition to the primary sRNA_162_ transcript and its respective 5′ processing fragments, other fragments were also detected using the probe directed against sRNA_162_ as evident in a sRNA_162_ deletion strain (Figure 1C). These are probably due to unspecific cross-hybridization (marked *) or cross-hybridization with sRNA_171_. To discriminate between the two homologous sRNAs, we additionally generated riboprobes specific for the respective full length sRNAs by *in vitro* transcription resulting in neglectable cross-hybridization in Northern blots (Supplementary Figure S1B and C). Hybridization with these riboprobes demonstrated that sRNA_171_ is also constitutively expressed and appears to be immediately processed into stable 5′ fragments, especially when cells enter stationary phase. However, additional processing fragments were detectable of ∼150 and 120 nt in length (Supplementary Figure S1C), indicating different processing of the two RNA species and/or different stabilities of the resulting 3′ fragments, though high conservation of their primary sequence (Figure 1D).

**Figure 2.**
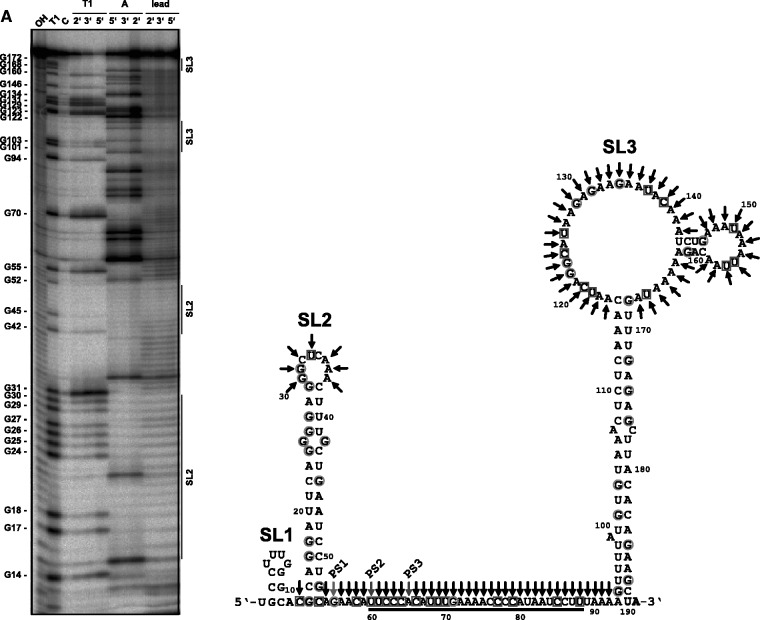
Structure mapping of 5′-end-labeled sRNA_162_ and proposed secondary structure of sRNA_162_. (**A**) 5 nM 5′-end-labeled sRNA_162 _was subjected to RNase T1, lead(II) and RNase A cleavage. The cleavage was performed for 2, 3 or 5 min, respectively. Lane OH: alkaline hydrolysis laddar. Lane T1: RNase T1 laddar under denaturing conditions. The position of the cleaved Gs is given on the left of the gel. Lane C: untreated sRNA_162_. The approximate positions of stem-loop structures SL1, SL2 and SL3 according to the sRNA_162_ structure shown in (**B**) are depicted by vertical bars on the right of the gel. (B) Proposed secondary structure of sRNA_162_ determined by *in vitro* structure mapping. Cleavages according to (A) are indicated by circles for RNase T1 cleavage under both native and denaturing conditions, by squares for native RNase A cleavage and by black arrows for native lead(II) cleavage. The 3′ ends of the three small processed fragments of sRNA_162_ detected by northern blot analysis are indicated as well as the predicted interacting site with MM2441 (black bar). Processing sites (PS1-3) are indicated.

BLAST searches for sRNA_162 _homologs in other prokaryotes detected the presence of homologous sequences exclusively in the two close relatives, *M. acetivorans* and *M. barkeri*. Secondary structure alignments of all sRNA_162_ homologs revealed not only highly conserved structural elements [stem loop (SL) 1–3] but also a 39 nt single stranded linker region (SLR) between SL2 and SL3 (Figure 1D), representing the most variable part within the predicted structural alignment. *In vitro* secondary structure probing using RNase T1, RNase A- or PbII-cleavage of *in vitro* synthesized 5′-end-labeled sRNA_162_, confirmed the *in silico* predicted secondary structure with only marginal variations in the organization of internal loops of SL3 (compare Figures 1E and 2). Analysis of the homologous sRNA_171_ revealed a similar secondary structure consisting of three stem loops (SL1–SL3), with the two structured elements SL2 and SL3 interrupted by a SLR of 39 nt (Supplementary Figure S2). Single nucleotide exchanges in comparison with sRNA_162 _are mostly present in the 5′ part of sRNA_171_ ranging from position 1 to 80. Of those located in the structured parts, the majority represents compensatory base-pair exchanges or nucleotide exchanges within bulge loops which do not affect base-pairing [e.g. G42 (sRNA_162_), A42 (sRNA_171_)]. Changes downstream of SL2 tend to be A to U substitutions, whereas at the cognate positions of sRNA_162_ Gs and Cs are dominating (Figures 1D and 2; Supplementary Figure S2).

### Identification of potential sRNA_162 _targets by genetic and computational approaches

A deletion mutant of sRNA_162 _and a mutant expressing the sRNA_162_ gene from the low copy plasmid pWM321 under the control of its native promoter (further designated as ‘over-expressing mutant’) were generated (see ‘Materials and Methods’ section). Northern blot analysis confirmed deletion of sRNA_162_ and showed ∼30-fold higher transcript levels in the over-expressing mutant strain (Figure 1C). To identify potential target genes of sRNA_162_, genome-wide changes in transcript levels in this mutant in comparison with the wt strain were studied using established genomic microarrays for *M. mazei* (41,45). The transcriptome analysis demonstrated that approximately 185 of the ORFs showed significantly different transcript levels (≥3-fold) in the over-expressing mutant compared with the wt strain. These include 48 genes involved in energy and constructional metabolism, two transcriptional regulators, 14 genes encoding transport or membrane proteins and additional 51 genes with unknown function (Supplementary Table S3). Strikingly, elevated transcript levels of a high number of genes (7 operons) encoding soluble methyltransferases involved in degradation of methylamines were obtained in the mutant, although the strains were grown on methanol (Table 1). A transcriptome analysis of the sRNA_162_ deletion mutant was not performed. Because the first 65 nt of sRNA_162_ overlap with the 5′UTR of the flanking gene MM2442, we assumed polar effects in the deletion mutant on MM2442 (discussed later, second target).

**Table 1.** Transcript levels of selected genes involved in TMA degradation and MM2441 of *M. mazei* sRNA_162_-overexpressing mutant versus *M. mazei* wt during growth on methanol as sole carbon and energy source identified by global expression profiling using genomic microarrays (41,45)

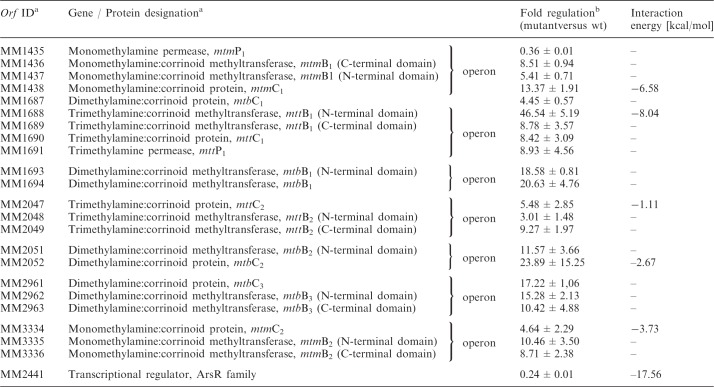

The *in silico* predicted interaction energy with sRNA_162_ is given.

^a^As defined in (54).

^b^Gene induction is represented as the ratio of median. Mean values obtained from five microarray experiments that satisfy the criteria defined in the ‘Material and Methods’ section are indicated.

**Figure 3.**
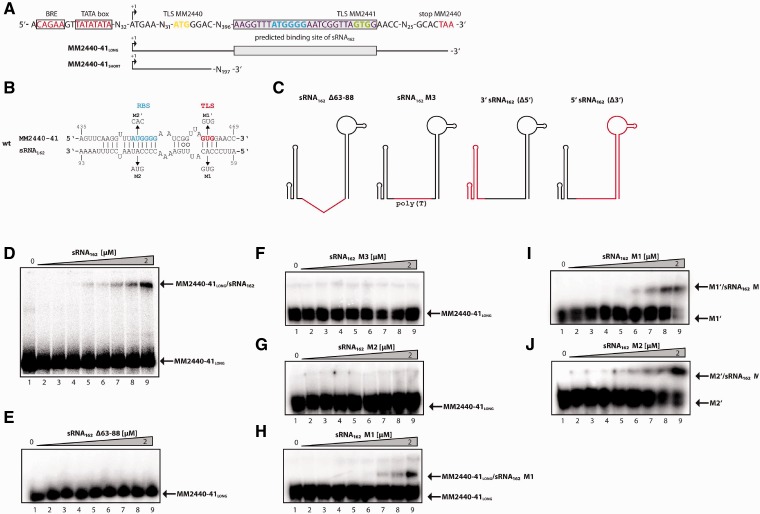
sRNA_162_ interacts with the bicistronic mRNA MM2440-41 *in vitro*. (**A**) Overview of the transcriptional and translational features of MM2440-41. The boxed region indicates the predicted interacting site. Bold letters either represent the RBS or TLS of the respective ORFs. The *in vitro* transcribed target mRNAs are schematically drawn below. (**B**) *In silico* predictions of the sRNA_162_–MM2440-41 interaction performed with IntaRNA (49). The wild-type (wt), triplet mutations (M1 and M2) and compensatory mutations (M1′ and M2′) are indicated. (**C**) Cartoon of the proposed structure of sRNA_162_. Deletions or replacements of sRNA parts are indicated in red. (**D–J**) Electrophoretic mobility shift assays were performed using approximately 5 nM of radioactively 5'-end-labeled MM2440-41_LONG_ (or compensatory mutants, respectively). The assays were performed as described in the ‘Materials and Methods’ section with increasing concentrations of unlabeled sRNA_162_ or mutated species from 0 to 2 µM. After 15 min incubation, samples were run on a native 6% PAA gel. The respective autoradiographs of the gels are shown.

All ORFs with significant changes in transcript levels in the overexpression strain (≥3-fold) were further analysed *in silico* to predict potential direct targets of sRNA_162_. The tool IntaRNA (49) was used to predict putative interactions between sRNA_162_ and each of the respective mRNAs, including either their native 5′UTR, if known (36), or if not known, including 200 nt upstream of the predicted translational start site (TLS) (see ‘Materials and Methods’ section). The obtained results for selected target candidates are summarized in Table 1. Additional potential interactions are listed in Supplementary Tables S4 and S5. The best scoring target was MM2441 encoded in reverse orientation upstream of the sRNA_162_ gene (Figure 1A). The predicted interaction between the two RNAs is centered on the potential RBS and the TLS of mRNA2441 as depicted in Figures 3A and B with a free energy score of −17.6 kcal/mol. This score is considerably better than the second best ranked target (MM2339) with a free energy score of −10.9 kcal/mol (Supplementary Table S4), or than any of the other methyltransferase genes with altered expression upon sRNA_162_ overexpression (see above and Table 1). Thus, the predicted primary target MM2441 was selected for further biochemical validation.

### *In vitro* verification of a direct sRNA_162_/mRNA 2441 interaction

Transcript analysis by 5′-RLM RACE revealed that MM2441 is encoded as the second gene in a bicistronic operon together with MM2440 (encoding a hypothetical protein), which is preceded by a short 5′-UTR of 36 nt (Figure 3A). Furthermore, the two ORFs overlap because the predicted TLS of MM2441 is located within the 3′ coding region of MM2440, 37 nt upstream of the translational stop of MM2440 (Figure 3A). Electrophoretic mobility shift assays using a 5′ labeled *in vitro* synthesized fragment of the bicistronic mRNA MM2440-41 (+1 to +469 relative to the TSS) including the predicted binding site (MM2440-41_LONG_; Figure 3A) and *in vitro* synthesized non-labeled full length sRNA_162_ (see ‘Materials and Methods’ section), demonstrated that binding to 5 nmol MM2440-41_LONG_ occurred at concentrations ≥32 nM of sRNA_162_ (Figure 3D). The reverse assay, using 5′-labeled sRNA_162_ and non-labeled MM2440-41_LONG_, further verified a direct interaction between the two RNAs (Supplementary Figure S4A). The interaction was strongly diminished when equal amounts of non-labeled sRNA_162_ were used as competitor RNA, confirming specificity of the binding (compare lines 9 and 10, Supplementary Figure S4A). In contrast, up to 2 µM of sRNA_162_ did not affect the mobility of a shorter 5′ fragment of the target mRNA, which did not include the predicted binding site (MM2440-41_SHORT_, ranging from+1 to 248; Supplementary Figures S4B and C), demonstrating that the predicted interaction site is compulsory for binding. The absence of the predicted, non-structured binding site in sRNA_162_ (sRNA_162Δ63-88_) or changing the region into polyT (sRNA_162_ M3), resulted in a complete loss of the mobility shift of MM2440-41_LONG_ (Figures 3E and F; reverse assays Supplementary Figure S4D and E). Triple point mutations within the potential interacting site changing either position 64–66 in sRNA_162_ from CAC antistart codon to GUG (M1 mutant) or position 79–81 from CAU to GUA (M2 mutant), further demonstrated that the M2 mutation totally abolished binding (Figure 3G; reverse assay Supplementary Figure S4F), whereas binding of the M1 mutant is only slightly reduced (Figure 3H and Supplementary Figure S4G reverse assay). Introducing compensatory mutations at position 461 to 463 (M2′) or 446 to 448 (M1′) of MM2440-41_LONG_, totally restored the interaction with the M2 mutant sRNA, whereas the interaction with sRNA_162_ M1 is significantly improved (Figure 3I and J; Supplementary Figure S4H and I). These findings strongly indicate a specific binding of sRNA_162 _to the mRNA (MM2440-41), which most likely leads to masking of the RBS and the TLS of MM2441, as depicted in Figure 3B (wt). The binding affinity appears to be relatively low in comparison with a variety of bacterial *trans*-acting sRNAs (e.g. GcvB; (4)), indicating that a chaperone such as Hfq might be required to facilitate annealing of the two RNAs. Thus, the effect of Lsm-like proteins (MM339, MM2383) on binding has been tested by gel shift experiments in the presence of up to 75 µM purified his-tagged proteins (calculated for the hexamers); however, no significant effect was observed (data not shown).

Gel shift assays using the 5′ fragment (nt 1–65) of sRNA_162 _(resembling the largest detectable stabile 5′ fragment *in vivo*) or the 3′ fragment (nt 65–191) containing most of the binding site (nt 60–88) but missing the structured loops SL1 and 2 (Figure 3C) further demonstrated, that both of the truncated fragments are not able to interact with the target mRNA (Supplementary Figure S4J–M). Because both mutants lack parts of the binding site, this strongly suggests that the entire interacting site is essential for effective target binding, and/or the structured 5′ part of sRNA_162_ (SL1 and 2), though not directly involved in the interaction, is highly required for a stable mRNA MM2440-41 target interaction *in vivo*.

### *In vivo* target validation by genetic approaches

**Figure 4.**
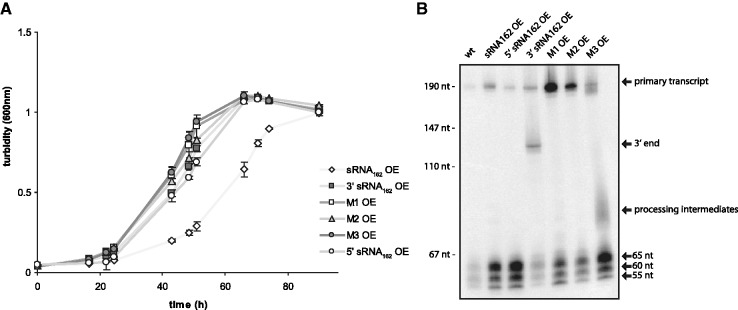
Growth and northern blot analysis of sRNA_162_ overexpressing mutants. (**A**) Growth of wt with additional sRNA_162_ (sRNA_162_ OE; empty diamond), 5' end of sRNA_162_ (5' end; empty circles), 3' end of sRNA_162_ (3' end; filled squares), M1 mutant (M1; empty squares), M2 mutant (M2; filled triangles) and M3 mutant (M3; filled circles), each ectopically overexpressed from pWM321 in wt background. Cells were grown on methanol to mid-exponential phase. (**B**) Northern blot analysis of the respective *M. mazei *strains using *in vitro* synthesized full-length RNA probes.

The mutant overexpressing sRNA_162_ showed a partial growth defect when grown on methanol as sole carbon and energy source (Supplementary Figure S5). To gain a deeper insight, whether the growth reduction is due to interaction of sRNA_162_ with the bicistronic operon MM2440-41, or due to the interaction with a yet unknown target (or a combination of both), additional mutant strains were generated. Mutant derivatives of sRNA_162_ identical to the variants used for EMSAs (Figure 3C), were ectopically expressed in the wt background from pWM321 under the control of the native promoter and with the native terminator, including the separated 5′ (1–65 nt) and 3′ fragment (65–191 nt, containing most of the predicted binding site), and the M1 to M3 derivatives of sRNA_162_. Neither overexpression of the fragments nor the mutant derivatives of the full length sRNA_162_ effected growth (Figure 4A); solely overexpressing full length wt sRNA_162_ led to the observed significant change in growth rate. This finding strongly indicates that exclusively the primary transcript, containing the intact wt SLR, is capable to interact with the proposed target(s) and is crucial for the obtained growth defect, most likely due to down regulation of MM2441. However, at the current experimental status we cannot rule out the possibility that the phenotype is due to interactions with other potential targets, since the chromosomal compensatory mutations in MM2440-2441 nor a double deletion of sRNA_162_ and MM2441 have not been generated due to experimental bottle necks (e.g. the lack of a second selection marker).

To test whether processing of the sRNA is altered in case of the sRNA_162_ derivatives northern blot analysis was performed (Figure 4B). Compared with the wt background, overexpression and subsequent processing of full length sRNA_162_ results in significant higher transcript levels of the primary transcript, as well as the three 5′ fragments. A similar increase of the three 5′ fragments was also detected upon overexpression of the 5′ fragment (1–65 nt) of sRNA_162_ (Figure 4B, lane 3) indicating that this extended 5′ fragment is further processed into similar fragments and ratio as for the processing of the full length sRNA_162_. Remarkably, overexpression of the 3′ fragment (nt 65–191), results in stable expression of the 3′ portion without detectable processing pointing toward a crucial role of the structured 5′ end of sRNA_162_ for processing. However, the increased stability of the 3′ fragment might also result from the artificial truncation. Mutant derivatives of full length sRNA, M1 and M2, showed higher accumulation of the primary transcript compared with the wt, strongly indicating altered processing. In contrast, when replacing the SLR by poly(T) (derivative M3), the primary transcript appears less stable compared with the M1 and M2 derivatives and the defined 5′ fragments are still produced in high amounts, although as well some additional processing products are visible (Figure 4B).

**Figure 5.**
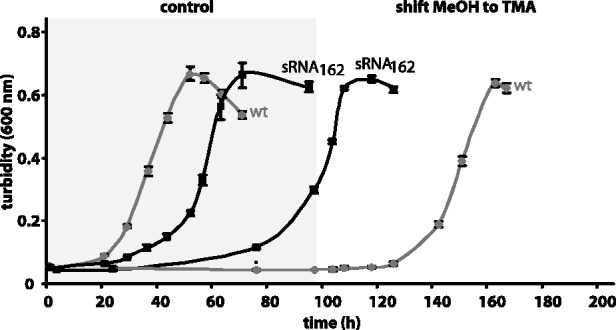
Growth behavior of the mutant overexpressing sRNA_162_ when shifted from methanol to trimethylamine in comparison with the wt. Cells were precultured as described in the ‘Materials and Methods’ section with methanol as sole carbon and energy source. After reaching exponential growth phase, cells were inoculated in fresh media either containing methanol (control) or trimethylamine (shift) as carbon and energy source. Growth was monitored by measuring the turbidity at 600 nm. Wt; sRNA_162_ OE, sRNA_162_ overexpressed from pWM321 in wt. Depicted are mean values and standard deviations of three biological replicates.

**Figure 6.**
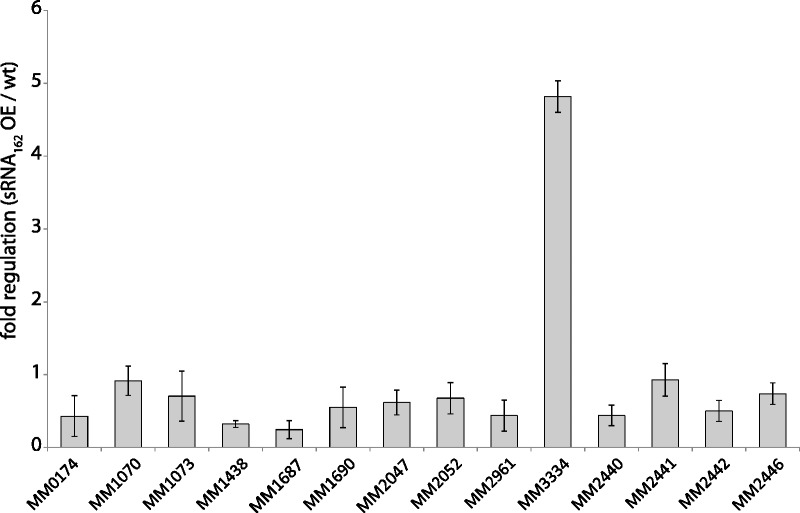
Transcriptional profile of selected genes in exponential growth phase. For selected genes, the transcriptional levels were determined in exponential growth phase by qRT-PCR (for primers see Supplementary Table S5). Fold changes in the sRNA_162_ overexpressing mutant (vs. wt) are given by mean values and standard deviation of three biologically independent experiments. MM0174, *mta*C_1_ encoding methanol corrinoid protein; MM1073, *mta*C_2_ encoding methanol corrinoid protein; MM1070, *mta*A_1_ encoding methylcobalamin-coenzyme M methyltransferase; MM1438, *mtm*C_1_ encoding monomethylamine corrinoid protein; MM1687, *mtb*C_1_ encoding dimethylamine corrinoid protein; MM1690, *mtt*C_1_ encoding dimethylamine corrinoid protein; MM2047, *mtt*C_2_ encoding trimethylamine corrinoid protein; MM2052, *mtb*C_2_ encoding dimethylamine corrinoid protein; MM2961, *mtb*C_3_ encoding dimethylamine corrinoid protein; MM3334, monomethylamine corrinoid protein; MM2440, hypothetical protein; MM2441,*: *transcriptional regulator, ArsR family; MM2442, hypothetical protein; MM2446, conserved protein.

Considering that several soluble methyltransferases involved in degradation of methylamines showed elevated transcript levels upon sRNA_162_-overexpression (Table 1), growth was studied with trimethylamine (TMA) as sole energy and carbon (C) source. No difference in expression or processing of sRNA_162_ was obtained when cells were grown on TMA (Supplementary Figure S1A). Moreover, when grown on TMA the sRNA_162_ overexpression strain displayed the same growth phenotype as observed with methanol (data not shown). However, when shifting cells from methanol to TMA, sRNA_162_-overexpression appears to result in significant faster adaptation following the C source shift compared with the wt (Figure 5) probably due to already synthesized soluble methyltransferases. To further support this suggestion, transcript levels of soluble methyltransferases in exponential growth phase with methanol were evaluated. Because of high sequence similarities of methyltransferase genes reliable transcript quantification using the PCR-fragment based microarray is not possible (45); thus, we performed qRT-PCR analysis using specific primers (46,55). A variety of methyltransferases with different substrate specificities showed significantly reduced mRNA levels of their respective operons, including methanol-dependent methyltransferases (*mta*C_1_B_1_, MM0174-75; *mta*C_3_B_3_, MM1648-47), TMA-dependent methyltransferases (*mtt*B_1_C_1_ MM1688-90; *mtt*B_2_C_2_, MM2049-47), dimethylamine-dependent methyltransferases (*mtb*C_1_B_1_, MM1687, 1693-94; *mtb*C_2_B_2_, MM2052-50) and monomethylamine-dependent methyltransferases (*mtm*C_1_B_1_, MM1438-36) (Figure 6 and Supplementary Figure S6), explaining the reduced growth rates on methanol and TMA. Solely, *mtm*C_2_B_2_, encoding a monomethylamine-dependent methyltransferase (MM3334-36), showed approximately 5-fold increased transcript levels upon sRNA_162_-overexpression, most likely leading to the observed faster adaptation of the sRNA_162_ overproducing mutant strain after a shift from methanol to TMA (Figure 6).

On the basis of these findings, we hypothesize that MM2441 is most likely post-transcriptionally regulated by sRNA_162 _and encodes a transcriptional regulator, which represses transcription of *mtmB_2_C_2_* encoding an essential soluble methyltransferase involved in TMA degradation. However, transcription of several other methyltransferase genes appears to be activated by MM2441 (see discussion).

### MM2442 as the second potential target of sRNA_162_

**Figure 7.**
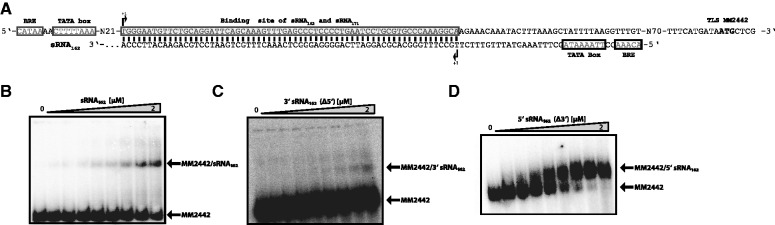
The 5'-UTR of MM2442 as a sRNA_162_ target. (**A**) Promotor region of MM2442 and sRNA_162_. The 5′ end (+1) of MM2442 was determined by 5′-RLM RACE. The TATA box and BRE are indicated for both, MM2442 and the *cis*-encoded antisense sRNA_162_. The predicted interacting site is boxed. (**B–D**) Electrophoretic mobility shift assays were performed using ∼5 nM of radioactively 5'-end-labeled MM2442. The reactions were performed as described in the ‘Materials and Methods’ section with increasing concentrations of unlabeled sRNA_162_ or mutated species from 0 to 2 µM. After 15 min incubation, samples were run on a native 6% PAA gel. The respective autoradiographs of the gels are shown.

Although short UTRs or even leaderless mRNAs have been predominantly found in (hyper-) thermophilic archaea (35,56), in methanoarchaea long 5′ UTRs with an average size of ∼150 nt are more the rule than the exception (36). Because sRNA_162_ is located 150 nt upstream of the TLS of MM2442 in opposite orientation (Figure 1A), we determined the TSS of MM2442 encoding a conserved protein of unknown function. 5′-RLM RACE analysis identified the TSS of MM2442 to be 184 nt upstream of the predicted TLS resulting in an overlap of 65 nt in antisense orientation with the 5′ end of sRNA_162 _(Figure 7A). This strongly suggests that besides targeting mRNA2440-41 in *trans*, sRNA_162_ very likely represents also a *cis*-encoded antisense regulator of MM2442 mRNA. Gel mobility shift experiments with full length sRNA_162 _and its 3′ and 5′ fragments demonstrated that full length sRNA_162_ indeed binds with its 5′ region to the 5′-UTR of MM2442 mRNA, whereas the isolated stable 5′ fragment appears to have even a significant higher binding affinity (Figure 7B–D; reverse assays Supplementary Figure S7). Moreover, *in vitro* foot printing analysis confirmed that sRNA_162_ binds to the leader of MM2442 by its first 65 nt as depicted in Supplementary Figure S8.

### Characterizing sRNA_171_ and its potential targets

The homologous sRNA_171_ is encoded in the IGR between MM2448 encoding a transposase and MM2449, encoding a protein of unknown function, which however shows some homology to MM2442. Upstream of MM2448 a second transposase gene (MM2447) is present, which is further upstream flanked by MM2446, encoding a transcriptional regulator of the ArsR family with MM2441 as the closest homolog (Supplementary Figure S9). Overall, the high conservation of the two sRNAs (including their promoters) as well as the conservation of the flanking regions of sRNA_162_ and sRNA_171_ and their respective genomic organization suggests that sRNA_171_ has been generated by a duplication of the complete gene locus MM2440–MM2442 most likely via a transposition event (Supplementary Figure S10). This is further supported by the finding that in the close relatives *M. acetivorans* and *M. barkeri* only a single copy of the sRNA is present in similarly organized genetic loci (Supplementary Figure S10).

Even though sRNA_171_ generally showed high sequence conservation to sRNA_162_ 4 nt changes are obvious within the SLR, which might alter the binding properties of the sRNA (Figure 1D). However, due to the similar genomic organization potential cross interaction between sRNA_171_ and the respective target mRNAs of sRNA_162 _and vice versa were investigated by *in silico* predictions (Supplementary Figure S9C) and gel shift experiments. This demonstrated a significantly lower binding affinity of sRNA_171_ to the 2440–41 mRNA than observed for sRNA_162_ (Supplementary Figure S9D; reverse assay Supplementary Figure S7) and no binding to MM2446 for both sRNAs (Supplementary Figure S9F and G; reverse assays Supplementary Figure S7). Most interestingly, the second target of sRNA_162_, the 5′ UTR of MM2442, was bound by sRNA_171_ (Supplementary Figure S9E; reverse assay Supplementary Figure S7) with similar affinity as observed for the 5′ fragment of sRNA_162_ (Figure 7D). Consequently, the targets of both sRNAs generated by duplication might formerly have been the same, but following the transposition event both sRNA-genes and targets might have picked up several different mutations leading to diversification of targets and functions.

## DISCUSSION

In the last two decades, a continuously growing number of sRNAs has been identified in prokaryotes. However, most of the functional characterization of sRNAs has been carried out in enterobacteria. Although expression of sRNAs has also been verified in several archaeal species (32–36,57–63), neither a potential target mRNA nor a molecular mechanism of target regulation or physiological role has been elucidated in archaea so far. Considering that cellular processes in archaea in general show many mechanistic features more similar to their eukaryotic than bacterial counterparts, e.g. transcription and translation machineries (64–68), 3′ targeting of mRNAs similar to eukaryotic miRNAs has been considered to be very likely for archaeal sRNAs. In this study, however, we present the first detailed characterization of an archaeal sRNA interacting with the 5′ UTR of two mRNAs, a *cis*- and a *trans*-encoded target.

### An archaeal sRNA masks the ribosome-binding site of its target

We provide several lines of evidence, that sRNA_162_ acts in *trans* on the neighboring operon MM2440-41 by internally binding to the bicistronic mRNA. The interacting site of sRNA_162_ was narrowed down to the SLR between SL2 and 3 (Figure 2) by *in vitro* binding assays and *in vivo* studies, clearly demonstrating that the SLR is crucial for functionality (Figures 3 and 4A). However, the observed binding affinity of sRNA_162_ to its target MM2441 is comparably low (for comparison see refs. 4, 69, 70). No further enhancement was obtained in the presence of the two heterologously expressed Lsm-like proteins of *M. mazei*, although we currently cannot exclude non-physiological folding or incorrect assembly of Lsm oligomers due to heterologous expression. The finding that sRNA_162_ binds to the predicted RBS as well as the TLS of MM2441, strongly argues that the interaction inhibits translation initiation most likely resulting in dis-coordinated operon expression. Because we do not see any changes at the mRNA level, regulation probably most likely occurs at the translational level. The discovery that sRNA_162_ targets the RBS within the 5′ region of MM2441 mRNA elucidated an unexpected regulatory mechanism of an archaeal sRNA, which was up to now exclusively described for bacterial sRNAs.

Homologs of RNase E and RNase III that are frequently involved in sRNA-mediated target regulation in bacteria (71–75) are not yet described in the archaeal domain. However, it is tempting to speculate that the increased turnover of sRNA_162_ into the stable 5′ fragments observed in stationary phase (Figure 1C and D) might be facilitated by orthologous archaeal RNases. This growth-phase dependent processing of sRNA_162_ leads to the accumulation of high amounts of stable 5′ fragments, indicating that processing may result in another (regulatory) outcome. However, ectopical overexpression of both, the longest 5′ fragment (65 nt) and the 3′ fragment of sRNA_162_ (including most of the predicted binding site) does not affect growth as observed upon overexpression of the full length sRNA_162 _(Figure 4A), strongly suggesting an essential (stabilizing) effect of the 5′ fragment on sRNA_162_, which appears crucial for functionality. Furthermore, it remains to be shown whether the resulting 5′ fragments of sRNA_162_ have additional regulatory functions.

### MM2441 most likely participates in the metabolic switch from methanol to (tri)methylamine

*In vivo* evidence was obtained that sRNA_162_ is involved in the adaptation process in response to different C-sources (e.g. shift from methanol to trimethylamine). Several soluble methyltransferases involved in methanol and methylamine-driven methanogenesis have been demonstrated to be differentially expressed upon overexpression of sRNA_162_ but were not predicted as direct targets by bioinformatic predictions (see Supplementary Tables S3–S5). Based on our finding that sRNA_162_ most likely interferes with MM2441 translation, we hypothesize that the indirect down-stream effects on transcription of several soluble methyltransferase genes in response to sRNA_162_ overexpression results from the reduced synthesis of the ArsR-type transcriptional regulator MM2441. We further propose that during growth on methanol MM2441 represses transcription of the MM3334-3335 operon, encoding a monomethylamine-dependent methyltransferase (*mtm*B_2_) and the cognate corrinoid protein (*mtm*C_2_). However, other methyltransferase operons [e.g. MM1438-34 (*mtm*C_1_B_1_P_1_) and MM1687, 1693-94 (*mtb*C_1_B_1_)] are most likely activated by MM2441. In line with this, significantly elevated transcript levels for the *mtm*B_2_C_2_ operon and reduced transcript levels for *mtm*C_1_ and *mtb*C_1 _were detected by qRT-PCR in the sRNA_162_ overexpressing mutant (Figure 6), probable due to decreased amounts of MM2441. This change in methyltransferase expression patterns upon sRNA_162_ overexpression most likely results in faster growth adaptation after a switch from methanol to TMA (Figures 6 and Supplementary Figure S6).

Soluble methyltransferase genes involved in the degradation of methanol and methylamines are in general among the most highly regulated genes in methanoarchaea (46,55,76–78). Several studies clearly demonstrated growth phase and carbon- and energy source dependent regulation of the respective enzymes in *M. acetivorans* and *M. mazei* and several overlapping regulatory circuits (55,76–79). A number of putative transcriptional regulators have been discovered, which impact on expression of the corresponding genes (76). For some of those, a dual functional role has been predicted, i.e. they act as activators for transcription of several methyltransferase genes as well as repressors for others (76), which is in agreement with our findings.

**Figure 8.**
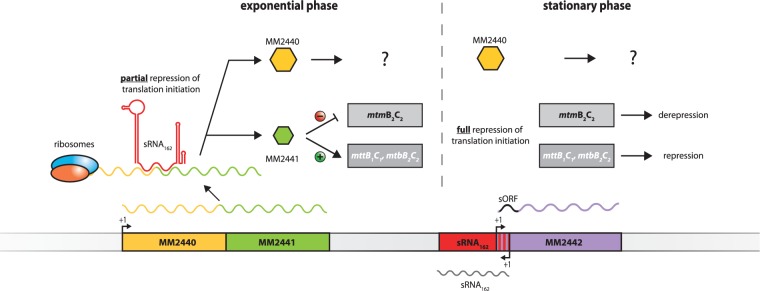
Proposed model of the sRNA_162_ regulatory network. (**A**) sRNA_162_ is constitutively expressed during exponential growth with methanol as sole carbon and energy source. When sRNA_162_ is transcribed, it subsequently binds to mRNA MM2440-2441, sequestering the RBS and TLS of MM2441, thereby controlling translation rates to ensure constantly low MM2441 levels. The low MM2441 protein levels are sufficient to repress the *mtm*BC_2_ operon expression. When the turnover of the primary transcript of sRNA_162 _is reinforced (e.g. in stationary phase or by overexpressing sRNA_162_) by a yet unknown factor, this results in enhanced translational repression of MM2441. At the same time, transcription of *mtm*B_2_C_2_ is de-repressed by reduced MM2441 protein levels. The 5′- UTR of MM2442 encodes two potential small oligopeptides (sORF, black)). The regulatory outcome of the interaction with sRNA_162_ is unknown.

Overall, sRNA_162_ appears to be involved in regulating the metabolic switch from methanol to methylamines during the transition of exponential to stationary growth phase, when cellular proteins and methylamines are degraded, most likely by post-transcriptional regulation of MM2441. We propose the following regulation (Figure 8): During exponential growth with methanol as carbon source, sRNA_162_ is constitutively transcribed and subsequently binds to mRNA MM2440-2441. Thereby, sRNA_162_ sequesters the translation initiation region (RBS and TLS) of MM2441, and controls its translation rates to ensure low MM2441 protein levels that are still sufficient to repress the *mtm*B_2_C_2_ operon expression. Upon binding to its target, sRNA_162_ might be simultaneously cleaved by RNases. However, in stationary growth phase the turnover of the sRNA_162 _primary transcript is reinforced by a yet unknown factor that reflects entering the stationary growth phase (or artificially by overexpressing sRNA_162_). This results in full translational repression of MM2441 and consequently de-repression of *mtm*B_2_C_2_ transcription as well as reduced transcription of other methyltransferase operons (e.g. *mtm*C_1_B_1_P_1_ and *mtb*C_1_B_1_) due to the absence of MM2441.

### sRNA_162_ interacts with *cis*- and *trans*-encoded targets via two distinct domains

Despite acting as a *trans*-encoded antisense (as)RNA targeting MM2441, we further demonstrated that sRNA_162_ also represents a *cis*-acting asRNA. While the SLR domain is essential for the proposed interaction with MM2441, we provided evidence that sRNA_162_ targets the 5′-UTR of MM2442 by its very 5′ end (nt 1–65). Because the transcript level of MM2442 is slightly negatively affected by sRNA_162_ overexpression (qRT-PCR, Figure 6), translational repression and subsequent degradation of the transcript appears most likely, but transcriptional inference or promoter occlusion by sRNA_162_ is also possible. Upstream of the TLS of MM2442 (encoding a protein of so far unknown function) no RBS is present. A more detailed inspection of the long 5′-UTR of MM2442, further demonstrated that the 5′ UTR sequence contains two small (s)ORFs, potentially encoding an oligopeptide of 23 or 30 amino acids (Supplementary Figure S11). This might indicate a coupled translation of the sORF and MM2442, and consequently, sRNA_162 _could act by targeting an upstream sORF of the bicistronic mRNA. Since sRNA_162_ is masking the preceding RBS and CDS of the putative oligopeptide, it could lead to simultaneous translational repression of the sORF and MM2442. A similar mechanism has been described for RyhB sRNA in *E. coli *(5), which represses the translation of the ferric uptake regulator (Fur) by binding to the RBS of a translationally coupled small ORF (designated as ‘uORF′) upstream of the Fur CDS. Moreover, translational activation of downstream genes linked to a sORFs is mechanistically also possible, as exemplified by PhrS sRNA in *Pseudomonas aeruginosa* (80). The accumulation of the sRNA_162_ 5′-fragments during stationary growth probably maintains or even enhances translational repression of MM2442 and the potential small peptide. However, ectopic overproduction of the 5′ fragment of sRNA_162 _did not result in an obvious phenotype. Therefore, the role of the sORF and MM2442 and the regulation by sRNA_162_ is still elusive and remains to be investigated.

Together these findings strongly suggest that sRNA_162_ acts on *cis*- and *trans*-encoded targets. Regulation of *trans*-encoded target mRNAs by *cis*-encoded asRNAs has been only proposed lacking any experimental evidence. Very recently Sayed *et al.* (81) described two RNAs organized as a type I toxin–antitoxin system consisting of SprA1, which encodes a small cytolytic peptide, and SprA1AS the *cis*-encoded riboregulator of SprA1 expression. Although SprA1AS overlaps with the 3′ end of SprA1 RNA, translation inhibition by SprA1 is achieved by a functional domain outside of the complementary target sequence by forming a pseudoknot (81). However, sRNA_162_, differs from this system as it is not encoded in the same genomic locus as its *trans*-encoded target mRNA, thus represents the first *cis*-encoded asRNA, additionally regulating a second target *in trans*.

In conclusion, we have demonstrated for the first time that an archaeal sRNA, sRNA_162_, has a regulatory function in archaea by interacting with the translation initiation region (RBS and / or TLS) of its *trans*-encoded target(s), indicating that this archaeal sRNA acts similar as its bacterial counterparts. Moreover, we obtained evidence that sRNA_162_ also targets a second, *cis*-encoded target. These obtained insights into the archaeal sRNA_162_ and its interaction with its targets blur the paradigm of a border between *cis*- and *trans*- encoded sRNAs.

## SUPPLEMENTARY DATA

Supplementary Data are available at NAR Online: Supplementary Tables 1–5 and Supplementary Figures 1–11.

## FUNDING

Funding for open access charge: German Research Council (DFG) priority program (SPP) 1258 ‘Sensory and Regulatory sRNAs in Prokaryotes’ [Schm1052/9-1, Schm1052/9-2].

*Conflict of interest statement*. None declared.

## Supplementary Material

Supplementary Data
