# Supplementary material for: An archaeal sRNA targeting cis- and trans-encoded mRNAs via two distinct domains
Source: Nucleic Acids Res. 2012 Sep 8;40(21):10964–79. doi: 10.1093/nar/gks847 (PMC3510493; doi:10.1093/nar/gks847)
Supplement: Supplementary Data [file supp_40_21_10964__index.html]

An archaeal sRNA targeting cis- and trans-encoded mRNAs via two distinct domains — An archaeal sRNA targeting cis- and trans-encoded mRNAs via two distinct domains — Supplementary Data 

# An archaeal sRNA targeting *cis*- and *trans*-encoded mRNAs via two distinct domains

## Supplementary Data

files

**Files in this Data Supplement:**

- Supplementary Data - pdf file
