# Supplementary material for: An archaeal sRNA targeting cis- and trans-encoded mRNAs via two distinct domains
Source: Nucleic Acids Res. 2012 Sep 8;40(21):10964–79. doi: 10.1093/nar/gks847 (PMC3510493; doi:10.1093/nar/gks847)
Supplement: Supplementary Data [file supp_gks847_nar-01234-c-2012-File002.pdf]

Supplementary:

"An archaeal sRNA targeting *cis*- and *trans*-encoded mRNAs via two distinct domains"

Dominik Jäger[1], Sandy R. Pernitzsch[1,2], Andreas Richter[3], Rolf Backofen[3], Cynthia M. Sharma[2], and Ruth A. Schmitz[1]

[1]Institut für Allgemeine Mikrobiologie, Christian-Albrechts-Universität zu Kiel, Am Botanischen Garten 1-9, 24118 Kiel, Germany

[2]Zentrum für Infektionsforschung, Universität Würzburg, Josef Schneider-Str. 2 /Bau D15, 97080 Würzburg, Germany

[3]Institut für Informatik, Albert-Ludwigs-Universität zu Freiburg, Georges-Koehler-Allee, 79110 Freiburg, Germany

Figure S1. Northern blot analysis of total RNA of wt and $sRNA_{162}$ mutant strains. RNA was isolated form early exponential phase (OD_{600} of 0.2, lanes 1), mid exponential phase (OD_{600} of 0.5, lanes 2) and stationary phase (OD_{600} of 0.7, lanes 3). (**A**) Northern blot analysis of $sRNA_{162}$ (oligoprobe) in cells grown on trimethylamine (TMA). (**B-C**) To discriminate between $sRNA_{162}$ and its homolog $sRNA_{171}$ Northern blot with full length RNA probes against $sRNA_{162}$ (B) and $sRNA_{171}$ (C) were performed. The three 5'-fragments of $sRNA_{162}$ and the primary transcript are indicated by arrows. Cross-hybridization of the $sRNA_{162}$ probe is indicated by an asterisk. The lower panel shows the expression of 5S rRNA of the respective RNA preparation. wt, wild type strain; $sRNA_{162}$ OE, $sRNA_{162}$ overexpressed from pWM321 in wt; and $\Delta sRNA_{162}$, chromosomal $sRNA_{162}$ deletion mutant.

Figure S2. **Structure mapping of 5'end labelled $sRNA_{171}$ and proposed secondary structure of $sRNA_{171}$.** (**A**) 5 nM 5'-end labelled $sRNA_{171}$ was subjected to RNase T1, lead(II) and RNase A cleavage. The cleavage was performed for 2, 3 or 5 min, respectively. Lane OH: alkaline hydrolysis laddar. Lane T1: RNase T1 laddar under denaturing conditions. Lane A: RNase A ladder. The position of the cleaved G's is given on the left of the gel. Lane C: untreated $sRNA_{171}$. The approximate positions of stem-loop structures SL1, SL2 and SL3 according to the $sRNA_{171}$ structure shown in (**B**) are depicted by vertical bars on the right of the gel. (**B**) Proposed secondary structure of $sRNA_{171}$ determined by in vitro structure mapping. Cleavages according to (**A**) are indicated by blue circles for RNase T1 cleavage under native, by red squares for native RNase A cleavage and by black arrows for native lead(II) cleavage.

Figure S3. **Hammerhead ribozyme fusion of $sRNA_{162}$.** **(A)** A DNA template for T7 polymerase was synthesized with the T7 promotor fused to an artificial hammerhead ribozyme sequence followed by $sRNA_{162}$ sequence. (**B**) Predicted folding into the ribozyme shape of the DNA template after transcription. The unpaired C between stem loop (SL) 1 and SL3 represents the autocatalytic cleavage site. Folding was predicted by RNAfold (83). (**C**) 6% denaturing PAA gel of the in vitro transcription reaction. The arrows indicate the respective products. SL, stem loop; HH, hammerhead ribozyme; M, RNA ladder.

Figure S4. **Gel retardation assays of $sRNA_{162}$ or derivates with mRNA MM2440-41 or compensatory mutants.** Electrophoretic mobility shift assays were performed using approximately 5 nM of radioactively 5'end labelled $sRNA_{162}$ or a mutated derivates. The reactions were performed as described in Materials and Methods

with increasing concentrations of unlabelled (m)RNA as indicated (and depicted in Fig. 3) from 0 to 2 µM. After 15 min incubation, samples were run on a native 6% PAA gel. An autoradiograph of the gels is shown.

Figure S5. **Growth of sRNA$_{162}$ mutant strains**. Growth of wt, wt/pWM321 (plasmid control), sRNA$_{162}$ overexpressed from pWM321 in wt (sRNA$_{162}$ OE) and sRNA$_{162}$ deletion mutant (ΔsRNA$_{162}$).

Figure S6. Operon structure and differential expression of methyltransferases in *M. mazei*. Differentially expressed genes are indicated in light or dark grey for mRNA levels detected by microarray- or qRT-PCR analysis, respectively. Cells were grown with methanol as sole carbon and energy source to mid exponential phase.

Figure S7. Gel retardation assays of sRNA$_{162}$ or sRNA$_{171}$ with MM2440-41$_{LONG}$, MM2442 or MM2446 mRNA. Electrophoretic mobility shift assays were performed using approximately 5 nM of radioactively 5'end labelled sRNA$_{162}$ (or a /mutated derviate) and sRNA$_{171}$. The reactions were performed as described in Materials and Methods with increasing concentrations of unlabelled MM2440-41 (E), MM2442 (A-C, F) or MM2446 mRNA (H & G) from 0 to 2 µM After 15 min incubation, samples were run on a native 6% PAA gel. An autoradiograph of the gels is shown.

Figure S8. Identification of sRNA$_{162}$ binding sites of MM2442 mRNAs by *in vitro* probing. (**A**) 5 nM 5'-end labelled MM2442 mRNA was subjected to RNase T1, lead(II) and RNase A cleavage. The cleavage was performed for 2, 3 or 5 min, respectively. Lane OH: alkaline hydrolysis laddar. Lane T1: RNase T1 laddar under denaturing conditions. Lane A: RNase A ladder. The position of the cleaved G's is given on the left of the gel. (**B**) 5 nM 5'-end labelled MM2442 mRNA was subjected lead(II) cleavage in the absence (-) or presence (+) of approximately 2µM of cold sRNA$_{162}$ RNA. The cleavage was performed for 2, 3 or 5 min, respectively.

Figure S9. Characterization of sRNA$_{171}$. (**A**) Genomic context of sRNA$_{171}$. (**B**) Promotor and terminator region of sRNA$_{171}$. The 5'-end (+1) of sRNA$_{171}$ was determined by 5'-RLM RACE, as well as the 3'-end (*) by 3'-RACE analysis. (**C**) IntaRNA predictions of sRNA$_{171}$ interactions with MM2440-41, MM2442 and MM2446. Additionally the predicted interaction for sRNA$_{162}$ and MM2446 is shown (**C** lower right panel). (**D-G**) EMSA experiemnts were performed as described. Here, ~5 nM 5'end-labelled target mRNA **(D)** MM2440-41; (**E**), MM2446; (**F** & **G)**, MM2446 were incubated with increasing amounts of sRNA$_{171}$ (**D-F**) or sRNA$_{162}$ (**G**). The respective autoradiographs of the gels are shown.

Figure S10. Genomic context of sRNA$_{162}$ and its homologs in *M. mazei* and related *Methanosarcina spec.*. The locus encoding sRNA$_{162}$ and its homologs is depicted in *M. mazei* and its close relatives *M. actetivorans* and *M. barkeri.* Unless otherwise stated, the respective colour codes indicate functional conservation of the genes. Red flags specify potential sRNA$_{162}$ (homolog) binding sites.

Figure S11. The 5'UTR of MM2442 potentially encodes for two small peptides. The promotor region and selected parts of MM2442 are depicted. The proposed ribosome binding site (RBS) is indicated in bold letters, whereas the binding site of sRNA$_{162}$ (sRNA$_{171}$) is boxed. Below, the amino acid sequence of the potential ORFs is shown. TLS, translational start site.

Table S1. Strains and plasmids used in this study

Strain/ plasmid	Genotype/description	Source/reference
M. mazei strain Gö1	Wild type	DSMZ No. 3647
*M. mazei**	potential cell wall mutant	(37)
M. mazei Δ$sRNA_{162}$	*M. mazei** Δ$sRNA_{162}$::pac, pur^R	This study
E. coli DH5α	general clonig strain	(84)
E. coli λpir JM109	general clonnig strain	(74)
**Plasmids**		
pBluescript SK^+	General cloning vector	Stratagene, La Jolla, US
pCR4-TOPO	General cloning vector for PCR products	Invitrogen, Darmstadt, GER
pMCL210	General cloning vector for PCR products	
pRS474	pWM321 ({Metcalf, 1997 #53}) containing $sRNA_{162}$ (IGR MM2441 –MM2442)	This study
pRS648	pBluescript SK+ containing ~ 1 kbp upstream fragment of $sRNA_{162}$	This study
pRS649	pBluescript SK+ containing ~ 1 kbp downstream fragment of $sRNA_{162}$	This study
pRS650	pBluescript SK+ containing up- and downstream fragments with pac-cassette replacing sRNA162 to construct chromosomal ΔsRNA162	This study
pRS765	pCR4-TOPO containing sRNA162 under the control of the T7 promoter	This study
pRS766	pCR4-TOPO containing sRNA162Δ63-88 under the control of the T7 promoter	This study
pRS699	pCR4-TOPO containing $sRNA_{162}$ (IGR MM2441 –MM2442)	This study
pRS700	pCR4-TOPO containing 3' end of sRNA162 (Δ5')	This study
pRS701	3' end of sRNA162 (Δ5') in pWM321	This study
pRS702	pCR4-TOPO containing M1 mutant of sRNA162	This study
pRS703	M1 mutant of sRNA162 in pWM321	This study
pRS704	pCR4-TOPO containing M2 mutant of sRNA162	This study
pRS705	M2 mutant of sRNA162 in pWM321	This study
pRS706	pCR4-TOPO containing M3 mutant of sRNA162	This study
pRS707	M3 mutant of sRNA162 in PWM321	This study
pRS708	pCR4-TOPO containing 5' end of sRNA162 (Δ3')	This study
pRS709	5' end of sRNA162 (Δ3') in pWM321	This study
pRS767	pCR4-TOPO containing MM2440-41 under the control of the T7 promoter	This study
pRS768	pCR4-TOPO containing MM2440-41 compensatory M1 mutant under the control of the T7 promoter	This study
pRS769	pCR4-TOPO containing MM2440-41 compensatory M2 mutant under the control of the T7 promoter	This study
pWM321	oriR6K, pC2A replicon, pur^R, bla^R	(38)
pRS207	Pac-cassette in pBluescript SK+	(37)

Table S2. Oligonucleotides used in this study

Designation	Sequence (5’ → 3’)	used for
**Mutant construction**		
s162-XhoI.for	CAT TAG CTC GAG ACA TTC CCT TTA ACT GAT C	Construction sRNA162-overexpression
s162-KpnI.rev	GAA TTC GGT ACC CAA CTC CAC TTG CAG TG	Construction sRNA162-overexpression
s162 Del1 XhoI	TTT CGC AAT ACT CGA GTT C	construction ΔsRNA162::pac
s162 Del2 EcoRI	CTT CGA TGT TTG GGA ATT CC	construction ΔsRNA162::pac
s162 Del3 EcoRI	GCC CAA AGG CAA GAA TTC AA	construction ΔsRNA162::pac
s162 Del4 XbaI	GTT TCT AGA TAT CCA TAT GTT CC	construction ΔsRNA162::pac
s162-Mut1-for	CTG CAG AAC ATT CCG TGA TTT GAA AAC CC	construction of sRNA162 M1
s162-Mut1-rev	GGG TTT TCA AAT CAC GGA ATG TTC TGC AG	construction of sRNA162 M1
s162-Mut2-for	TCC CAC ATT TGA AAA CCC GTA AAT CCT TTA AAA GTT T	construction of sRNA162 M2
s162-Mut2-rev	AAA CTT TTA AAG GAT TTA CGG GTT TTC AAA TGT GGG A	construction of sRNA162 M2
s162-Mut3-for	TTT TTT TTT TTT TTA AAA GTT TTA TGT GTA AAC	construction of sRNA162 M3
s162-Mut3-rev	AAA AAA AAA AAA AAG GGA ATG TTC TGC	construction of sRNA162 M3
s162-Mut4-for	GAA TTC GGT ACC CAA CTC CAC TTG CAG TG	construction of Δ3’ end of sRNA162
s162-Mut4-rev	TTT CTC GAG TGG GAA TGT TCT GCA GGA TTC	construction of Δ3’ end of sRNA162
s162-Mut5-for	AGA AAC AAA TTA CTT TAA AGC TAT TTT AAG	construction of Δ5’ end of sRNA162
s162-Mut5-rev	AT TTG AAA ACC CCA TAA TCC TT	construction of Δ5’ end of sRNA162
2441-com_Mut1for	GGA ATC GGT TAC ACG AAC CGA GGA CTA	construction of T7::MM2440-41 M1
2441-com_Mut1rev	TAG TCC TCG GTT CGT GTA ACC GAT TCC	construction of T7::MM2440-41 M1
2441-com_Mut2for	AAG CTG GGA GTT CAA GGT TTT ACG GGA ATC	construction of T7::MM2440-41 M2
2441-com_Mut2rev	GAT TCC CGT AAA ACC TTG AAC TCC CAG CTT	construction of T7::MM2440-41 M2
**Northern blot analysis**		
s162-NB	TGCCGGATATCTGACCTGGTC	Probe against sRNA162
s171-NB	GGA TTA TGA GTT TTT TGA ATG TGG	Probe against sRNA171
asPs162 for	TGC CTT TGG GCA CGC AGG ATT CAG GG	generate antisense full length riboprobe against sRNA162
asPs162 rev	GAA ATT AAT ACG ACT CAC TAT AGG GTA GCT TCT GTG TAA G	generate antisense full length riboprobe against sRNA162
asPs171 for	TGC CTT CGG GTA TGC AGG ATT CAG GGG	generate antisense full length riboprobe against sRNA171
asPs171 rev	GAA ATT AAT ACG ACT CAC TAT AGG GTA GCT TCT GTG TAA G	generate antisense full length riboprobe against sRNA171
**RACE-experiments**		
5’-RACE 2441	GTA TTC TTC TCT TAT GCC TGA TTG TTT GAG AGT TTA	5’end determination sRNA162
3’-RACE 2441 #2	CAC GCA GGA TTC AGG GGG CTC	3’end determination sRNA162
5’-2441-RLM-out	GGGCCAGCCACAGGAGGAAC	5’end determination MM2442
5’-2441-RLM-in	GAAGCTCCAGATTGATCGCTCGG	5’end determination

		MM2442
5'-RLM 2446-out	CCC TTG GAA ATT CCA ATC TCA CGC GC	5’end determination MM2446
5'-RLM 2446-in	GGA CTG TCC TCC TGT TCA AGC CAG	5’end determination MM2446
5'-RLM-2442-out	cCC CGT TGT CCA CAA CTT GTG	5’end determination MM2446
5'-RLM-2442-in	CTG CAG ACC CAT CTT CCA GTG	5’end determination MM2446
**Quantitative RT-PCR**		
MM0021for.rt	AAT TTC GGC GCT CCC AGA GAA	Conserved protein
MM0021rev.rt	TAG CGG TTT TCT CTG GGC TTC	Conserved protein
MM0345for.rt	TAT AGG ACT CCT GAG ACA GGG	Transcriptional regulator
MM0345rev.rt	CCT TTC TAA CAG CTG TTC CGG	Transcriptional regulator
MM0339for.rt	GGC AAA CCG ACC TCT GGA TAT	Lsm-like protein
MM0339rev.rt	ACC GCG AAT AAC GAC ACT GCT	Lsm-like protein
MM1138for.rt	AGC GCC CTT GGC ACT ATA GAA	Glycosyltransferase
MM1138rev.rt	TTT CCG TAT CCA CGC CGT TAG	Glycosyltransferase
MM1438for.rt/	GGG CTT GTG TCC AGC ATA AAC	mtmC1 – MMA corrinoid protein 1
MM1438rev.rt/	CCT CCG TTT CTG TCG AAA ACG	mtmC1 – MMA corrinoid protein 1
MM1688for.rt/	CTT ATT CGG AGG TTA CAA CCC TGG	mttB1 – TMA ,methyltransferase 1
MM1688rev.rt/	GAA TTG CTT TGA GTT CAT CAG TGG	mttB1 – TMA ,methyltransferase 1
MM1732for.rt	GGA CGC GAA TAT TTC CTG ACG	Transcriptional regulator, ArsR-family
MM1732rev.rt	ATG CCT CGA TCC TTG AGG ACT	Transcriptional regulator, ArsR-family
MM2049for.rt/	CCT GAA GCA AGG CAG ATC TTC	mttB2 – TMA methyltransferase 2
MM2049rev.rt/	CAG AGA ACG AAC CTT GAA GGC	mttB2 – TMA methyltransferase 2
MM2052for.rt/	TGG GGG TTG AGA ATG ACA AGC	mtbC2 – DMA corriniod protein 2
MM2052rev.rt/	AGG CTC CAT AAC CTG CTT TGC	mtbC2 – DMA corriniod protein 2
MM2230for.rt	TCC TTG CCT GTG AAC CGT TGA	transposase
MM2230rev.rt	AGA GAG ATC CAG GTA TGT GCG	transposase
MM2339for.rt	GAG GAT TCC GGG CTC CAA AAA	EIF 1A
MM2339rev.rt	GGT ACC CTT TGC GTT CAA GCC	EIF 1A
Mm2441for.rt	CTG CTG AAC CCT GCA AAG CCT	Transcriptional regulator, ArsR-family
Mm2441rev.rt	TAA CCG TTC CCC TGT TCA AGC	Transcriptional regulator, ArsR-family
MM2888for.rt	AAC TCC ACG GCA GCA TTG GAA	endonuclease
MM2888rev.rt	TTT CCG GGG AAA GTG AGT CCA	endonuclease
MM3197for.rt	GAC TCT TCT CCT TTA TGC CGG	Hypothetical protein
MM3197rev.rt	TAT CAC CAG CAG GAC AAA GCC	Hypothetical protein
MM3334for.rt/	GAA AGA AGT GAT TAT GGG ATG AC	mtmC2 – MMA corrinoidp 2
MM3334rev.rt/	AGA GGG GAA ATT CCT GCA TC	mtmC2 – MMA corrinoidp 2
MM1621for.rt	TAG GAG GTT TTC TCG GAA GCG	Cobalamin biosyntehsis protein CobW
MM1621rev.rt	AAG CGT ATC TCC ATC AAG CCC	Cobalamin biosyntehsis protein CobW
MM2181for.rt	GCC TCC ATG AGA AGA ATG CTC	Fructose-1,6-bisphosphatase
MM2181rev.rt	CTT CAA GGT CTC CAA CTC CTG	Fructose-1,6-bisphosphatase
MM1215for.rt	TCA AGA GCG AGG GCA TGA ATG	Hexulose-6-phosphat synthase
MM1215.for.rt	GCA CTA CCG AGA ACA ATA GCC	Hexulose-6-phosphat synthase
MM2442-qRT-for	GGA GTG GTG AAA AAC CTG CAC A	hypothetical protein
MM2442-qRT-rev	AGC TCA TTC GCA ACA CAG GTC	hypothetical protein
MM2449-qRT-for	GTT ACG CCT GCT ATG GCG T	hypothetical protein
MM2449-qRT-rev	CTT TCT TCG GTC TGG GGA TTG	hypothetical protein
MM2440-qRT-for	AGT TTC AGC CGA AGA GGA ATA	conserved protein
MM2440-qRT-rev	AGT TAT GTA CTG GGT TGA CAG	conserved protein

MM2446-qRT-for	GAA TAC ATG TCA GTC CCT GG	transcriptional regulator, ArsR family
MM2446-qRT-rev	TAA ACG CTA TCT CGG TTA GG	transcriptional regulator, ArsR family
**Shift assay**		
T7-2440-41-for	GTTT TTT TAA TAC GAC TCA CTA TAG GGA AAA GAT GGA A	In vitro transcription MM2440-2441
T7-2440-41-rev1	CTT ACA TAG TGA GTT TTA GTC TGT CCT TGA G	In vitro transcription MM2440
T7-2440-41-rev2	GTG CTG GTA AAG ATT AAA TGT GTA GTC CTC	In vitro transcription MM2440-2441
T7-HH-s162-for	GAA ATT AAT ACG ACT CAC TAT AGG GTT TGG CAC TG	In vitro transcription sRNA162
sRNA162_T7.rev	TAG CTT CTG TGT AAG CTC TCA AAC TAT TTT TCT GTT	In vitro transcription sRNA162
T7-s162-short-for	GAA ATT AAT ACG ACT CAC TAT AGG GCA TTT GAA AAC CC	In vitro transcription sRNA162
T7::sRNA171-for	AAT TAA TAC GAC TCA CTA TAG GGC CTT CGG GTA T	In vitro transcription sRNA171
T7::sRNA171-rev	TAG CTT CTG TGT AAG CTC TCA AAT TAT TTT TCT G	In vitro transcription sRNA171
T7::2442-L-for	TAG ATT AAT ACG ACT CAC TAT AGG GAA TGT TCT GCA	In vitro transcription MM2442
T3-MM2446-for	GTT TTT TTA ATT AAC CCT CAC TAA AGG GTT CTG GTG CAA	In vitro transcription MM2446
T3-MM2446-rev	GGA CTG TCC TCC TGT TCA AGC CAG	In vitro transcription MM2446

Table S3. Different transcript levels of *M. mazei* $sRNA_{162}$-overexpressing mutant vs. *M. mazei* wild type strain during growth on methanol as sole carbon and engergy source identified by global expression profiling using genomic microarrays (45, 41)

***Orf* ID**[1]	**Gene /Protein designation**[1]	**fold regulation**[2] (mutant vs. wt)
	**Energy and constructional metabolism**	
MM2313	F_{420}-nonreducing hydrogenase I, large subunit	0.02 ± 0.01
MM0756	6-pyruvoyltetrahydropterin synthase	0.14 ± 0.00
MM1271	Fructose-bisphosphate aldolase	0.15 ± 0.00
MM1273	3-dehydroquinate synthase	0.15 ± 0.00
MM0626	Nicotinamide-nucleotide adenylyltransferase	0.15 ± 0.07
MM0964	Glutamine synthetase	0.16 ± 0.06
MM1272	3-dehydroquinate synthase	0.18 ± 0.01
MM2487	F_{420}-H_2 dehydrogenase, subunit H	0.18 ± 0.12
MM0299	Glycose-1-phosphate thymidylyltransferase	0.18 ± 0.15
MM1275	Prephenate dehydrogenase	0.19 ± 0.00
MM1274	Shikimate 5-dehydrogenase	0.19 ± 0.01
MM0627	F_{420}-H_2 dehydrogenase, subunit FpoF	0.19 ± 0.12
MM2488	F_{420}-H_2 dehydrogenase, subunit D	0.19 ± 0.13
MM2481	F_{420}-H_2 dehydrogenase, subunit M	0.20 ± 0.08
MM1544	Tetrahydromethanopterin S-methyltransferase, subunit B	0.23 ± 0.01
MM2093	Indolepyruvate oxidoreductase	0.23 ± 0.02
MM1547	Tetrahydromethanopterin S-methyltransferase, subunit E	0.24 ± 0.02
MM0242	Glycerol-3-phosphate cytidylyltransferase	0.25 ± 0.00
MM2321	Ech-hydrogenase	0.25 ± 0.01
MM2480	F_{420}-H_2 dehydrogenase, subunit N	0.25 ± 0.07
MM0628	Coenzyme F420-dependent N5-N10 methylen-tetrahydromethanopterin reductase	0.26 ± 0.09
MM2483	F_{420}-H_2 dehydrogenase, subunit K	0.26 ± 0.12
MM2322	Ech-hydrogenase	0.27 ± 0.02
MM2479	F_{420}-H_2 dehydrogenase, subunit O	0.28 ± 0.10
MM1062	Hydrogenase 4, compound F	0.29 ± 0.01
MM1279	Conserved protein (sugar metabolism)	0.29 ± 0.02
MM1545	Tetrahydromethanopterin S-methyltransferase, subunit C	0.29 ± 0.11
MM1059	Format hydrogen lyase, subunit 3	0.29 ± 0.14
MM1138	Glycosyltransferase	0.30 ± 0.00
MM0409	3-isopropylmalate dehydratase	0.30 ± 0.01
MM1238	3-phosphoshikimate 1-carboxyvinyltransferase	0.30 ± 0.01
MM1219	Fe-S oxidoreductase	0.30 ± 0.04
MM2323	Ech-hydrogenase	0.30 ± 0.14
MM0941	Adenosylosuccinate lyase	0.31 ± 0.00
MM1201	Dihydrodipicolinate synthase	0.31 ± 0.02
MM2320	Ech-hydrogenase	0.31 ± 0.07
MM1543	Tetrahydromethanopterin S-methyltransferase, subunit A	0.31 ± 0.10
MM1567	Molybdenum-formylmethanofuran dehydrogenase	0.31 ± 0.15
MM0855	Phosphoribosyl-aminoimidazol-succino carboxamide synthase	0.33 ± 0.07
MM0966	Glutamate synthase	0.33 ± 0.10

[1] as defined (81)
[2] Gene induction is represented as the ratio of median. Mean values obtained from 5 microarray experiments that satisfy the criteria defined in Material and Methods are indicated.

MM1602	Cobaltochelatase	0.33 ± 0.15
MM1435	Monomethylamine permease, *mtmP1*	0.36 ± 0.01
MM2048	Trimethylamine:corrinoid methyltransferase, *mttB2* (C-terminal domain)	3.01 ± 1.48
MM0166	Homospermidine synthase	3.09 ± 0.03
MM0514	Nitrogenase iron protein 2 nifH2	3.24 ± 0.13
MM1438	Monomethylamine:corrinoid protein, *mtmC1*	3.37 ± 1.91
MM1685	CDP-diacylglycerol-glycerol-3-phosphate 3-phosphatidyltransferase	3.63 ± 1.44
MM0689	CO-dehydrogenase/acetyl-CoA synthase, subunit γ	3.85 ± 0.74
MM1692	Conserved protein	3.91 ± 0.82
MM0686	CO-dehydrogenase/acetyl-CoA synthase, subunit β	3.93 ± 1.29
MM0999	Precorrin-2-C_{20} methyltransferase	4.03 ± 4.03
MM0387	Heterodisulfide reductase, subunit HDRA	4.04 ± 0.63
MM2085	CO-dehydrogenase/acetyl-CoA synthase, subunit δ	4.12 ± 0.89
MM0688	CO-dehydrogenase/acetyl-CoA synthase, subunit δ	4.24 ± 0.98
MM2089	CO-dehydrogenase/acetyl-CoA synthase, subunit α	4.26 ± 0.36
MM0134	Sulfopyruvate decarboxylase α-chain	4.29 ± 0.36
MM1737	Heme-biosynthesis protein	4.29 ± 1.69
MM1290	Fe-S oxidoreductase	4.42 ± 0.80
MM1978	Tungsten formylmethanofuran dehydrogenase	4.44 ± 0.22
MM1687	Dimethylamine:corrinoid protein, *mtbC1*	4.45 ± 0.57
MM2823	Indole-3-glycerol phosphate synthase	4.56 ± 0.31
MM0685	CO-dehydrogenase/acetyl-CoA synthase, subunit ε	4.63 ± 1.81
MM3334	Monomethylamine:corrinoid protein, *mtmC2*	4.64 ± 2.29
MM0388	Heterodisulfide reductase, subunit HDRC	4.76 ± 1.16
MM1025	Thiamine biosynthesis protein, *thiC*	4.79 ± 0.03
MM0389	Heterodisulfide reductase, subunit HDRB	5.04 ± 0.18
MM2084	Acetyl-CoA decarbonylase/synthase complex, subunit γ	5.20 ± 0.25
MM1437	Monomethylamine:corrinoid methyltransferase, *mtmB1* (N-terminal domain)	5.41 ± 0.71
MM2047	Trimethylamine:corrinoid protein, *mttC2*	5.48 ± 2.85
MM0684	CO-dehydrogenase/acetyl-CoA synthase, subunit α	6.16 ± 0.62
MM0133	Threonine synthase	7.79 ± 0.40
MM1690	Trimethylamine:corrinoid protein, *mttC1*	8.42 ± 3.09
MM1436	Monomethylamine:corrinoid methyltransferase, *mtmB1* (C-terminal domain)	8.51 ± 0.94
MM3336	Monomethylamine:corrinoid methyltransferase, *mtmB2* (C-terminal domain)	8.71 ± 2.38
MM1689	Trimethylamine:corrinoid methyltransferase, *mttB1* (C-terminal domain)	8.78 ± 3.57
MM2821	Tryptophan synthase, subunit α	8.81 ± 0.18
MM1691	Trimethylamine permease, *mttP1*	8.93 ± 4.56
MM2049	Trimethylamine:corrinoid methyltransferase, *mttB2* (N-terminal domain)	9.27 ± 1.97
MM0687	Nitrogenase iron protein	9.42 ± 0.88
MM1284	2-isopropylmalate synthase	9.80 ± 0.61
MM2963	Dimethylamine:corrinoid methyltransferase, *mtbB3* (C-terminal domain)	10.42 ± 4.88
MM3335	Monomethylamine:corrinoid methyltransferase, *mtmB2* (N-terminal domain)	10.46 ± 3.50
MM2051	Dimethylamine:corrinoid methyltransferase, *mtbB2* (N-terminal domain)	11.57 ± 3.66
MM2962	Dimethylamine:corrinoid methyltransferase, *mtbB3* (N-terminal domain)	15.28 ± 2.13
MM2961	Dimethylamine:corrinoid protein, *mtbC3*	17.22 ± 1,06
MM2820	Anthranilate phosphoribosyltransferase	17.25 ± 2.10
MM2817	Anthranilate synthase, component I	18.28 ± 1.20
MM1693	Dimethylamine:corrinoid methyltransferase, *mtbB1* (N-terminal domain)	18.58 ± 0.81
MM2818	Anthranilate synthase, component II	19.71 ± 0.38
MM1694	Dimethylamine:corrinoid methyltransferase, *mtbB1* (C-terminal domain)	20.63 ± 4.76

MM2052	Dimethylamine:corrinoid protein, *mtbC2*	23.89 ± 15.25
MM1688	Trimethylamine:corrinoid methyltransferase, *mttB1* (N-terminal domain)	46.54 ±5.19
	**Potential regulatory proteins**	
MM2441	Transcriptional regulator, ArsR family	0.24 ± 0.01
MM1332	Iron-dependent repressor	3.33 ± 0.22
MM1075	Putative regulatory protein	5.64 ± 0.21
MM3117	Transcriptional regulator, MarR family	7.85 ± 0.88
MM0732	Nitrogen regulatory protein II	7.95 ± 0.83
	**Transport/Membrane proteins**	
MM2305	Sodium/proline symporter	0.16 ± 0.01
MM3230	Phosphate permease	0.16 ± 0.03
MM3262	Aminoacid transport protein	0.23 ± 0.02
MM0002	Dipeptide ABC transporter-binding protein	0.23 ± 0.07
MM2576	Ferrous iron transport protein	0.33 ± 0.02
MM1553	Transporter, RND-superfamily	0.33 ± 0.07
MM0576	Phosphate transport system protein phoU	3.09 ± 0.21
MM2894	ABC transporter, ATP-binding protein	3.25 ± 0.67
MM0570	ABC transporter ATP-binding protein	3.26 ± 1.34
MM2457	Dipeptide/oligopeptide transporter, permease protein	3.29 ± 0.06
MM2459	Methyltransferases	3.34 ± 0.27
MM2387	Cobalt transport ATP binding protein, *cbiO*	3.34 ± 0.35
MM2833	ABC transporter, ATP-binding protein	3.70 ± 0.11
MM0834	Na^+/H^+ antiporter	3.79 ± 4.24
MM2455	ABC transporter, ATP-binding protein	4.53 ± 0.23
MM2458	Dipeptide/oligopeptide transporter, permease protein	5.34 ± 0.25
MM2460	Dipeptide/oligopeptide-binding protein	5.77 ± 0.06
MM2456	Dipeptide/oligopeptide transporter, ATP-binding protein	6.45 ± 0.25
MM2388	Colbalt transport protein, *chiQ*	7.42 ± 0.31
	**Diverse**	
MM2626	DNA-directed RNA polymerase	0.20 ± 0.01
MM0077	Icc protein	0.21 ± 0.11
MM2339	Translation initiation factor 1A	0.23 ± 0.05
MM2715	GTP-binding protein	0.24 ± 0.01
MM2268	Transcription elongation factor NusA-like protein	0.32 ± 0.00
MM2656	Peptidyl-prolyl cis-trans isomerase	0.32 ± 0.04
MM0190	DNA helicase I	3.01 ± 0.16
MM1306	HTH DNA-binding protein	3.03 ± 0.22
MM2584	Sec-independent protein translocase, protein tatA	3.51 ± 0.21
MM1362	Putative aliphatic sulfonate-precursor protein	3.79 ± 0.14
MM3037	Transposase	3.79 ± 2.86
MM2338	DNA-directed RNA polymerase I, II and III, 7.3 kDa polypeptide	3.84 ± 0.12
MM0102	Serine/threonine protein phosphatase	4.67 ± 4.98
MM3194	DinF protein	4.81 ± 0.02
MM0483	Small heat shock protein	11.94 ± 0.59
MM1236	Protease HTP	13.51 ± 0.27
MM3118	ATP-dependent protease L	14. 94 ± 0.68
	**Conserved and hypothetical proteins**	
MM0755	Hypothetical protein	0.13 ± 0.00

MM0804	Hypothetical ATP-binding protein	0.13 ± 0.01
MM0754	Hypothetical protein	0.14 ± 0.00
MM0021	Conserved protein	0.16 ± 0.06
MM1551	Hypothetical protein	0.20 ± 0.03
MM2531	Hypothetical protein	0.21 ± 0.01
MM0978	Hypothetical protein	0.22 ± 0.04
MM0758	Hypothetical protein	0.23 ± 0.00
MM1929	Hypothetical protein	0.23 ± 0.03
MM2182	Hypothetical protein	0.25 ± 0.02
MM0563	Hypothetical protein	0.25 ± 0.03
MM0229	Hypothetical protein	0.25 ± 0.08
MM1492	Hypothetical protein	0.26 ± 0.01
MM0827	Hypothetical protein	0.27 ± 0.00
MM0423	Hypothetical protein	0.27 ± 0.04
MM1433	Hypothetical protein	0.27 ± 0.04
MM0562	Hypothetical protein	0.28 ± 0.02
MM3130	Hypothetical protein	0.29 ± 0.01
MM3184	Conserved protein	0.29 ± 0.01
MM1676	Hypothetical protein	0.29 ± 0.02
MM0883	Hypothetical protein	0.29 ± 0.03
MM0774	Hypothetical protein	0.29 ± 0.04
MM0924	Conserved protein	0.29 ± 0.12
MM0386	Hypothetical protein	0.30 ± 0.02
MM1302	Hypothetical protein	0.30 ± 0.02
MM1793	Hypothetical protein	0.30 ± 0.02
MM2564	Hypothetical protein	0.30 ± 0.02
MM1125	Conserved protein	0.31 ± 0.00
MM0842	Conserved protein	0.31 ± 0.02
MM3345	Hypothetical protein	0.32 ± 0.01
MM1311	Hypothetical protein	0.32 ± 0.03
MM1116	Hypothetical protein	0.32 ± 0.06
MM1204	Hypothetical protein	0.33 ± 0.00
MM3306	Hypothetical protein	0.33 ± 0.09
MM1675	Hypothetical protein	0.34 ± 0.01
MM0753	Conserved protein	3.10 ± 1.04
MM2948	Hypothetical protein	3.34 ± 0.20
MM0138	Conserved protein	3.41 ± 0.00
MM3352	Hypothetical proetin	3.41 ± 0.01
MM1818	Conserved protein	3.41 ± 3.92
MM0095	Hypothetical protein	3.46 ± 0.07
MM2119	Hypothetical protein	3.68 ± 0.06
MM0925	Hypothetical protein	3.97 ± 0.20
MM3258	Hypothetical protein	4.19 ± 4.69
MM3193	Hypothetical protein	5.42 ± 0.87
MM0206	Conserved protein	5.54 ± 4.09
MM1674	Hypothetical protein	5.92 ± 0.97
MM0364	Hypothetical protein	6.01 ± 2.45
MM1294	Hypothetical protein	9.63 ± 0.57
MM3197	Hypothetical protein	10.54 ± 8.20

MM2860	Conserved protein	10.92 ± 14.71
MM0019	Hypothetical protein	14.04 ± 1.56

Table S4. Predicted interactions with $sRNA_{162}$ for ORFs with reduced gene expression upon $sRNA_{162}$overexpression. Interactions were analyzed using the IntaRNA tool (48). Predicted interaction site positions are given for the mRNA relative to the start codon and for $sRNA_{162}$. The interaction energy includes the interaction site, unfolding energies and the duplex energy.

**Target**	**Interaction site $sRNA_{162}$**	**Interaction site target**	**Interaction energy [kcal/mol]**
MM_2441	(63,88)	(-22,4)	-17,56
MM_2339	(61,86)	(3,25)	-10,88
MM_0978	(77,91)	(-7,8)	-9,98
MM_3121	(71,89)	(-11,8)	-9,02
MM_1547	(58,90)	(-16,27)	-8,89
MM_0745	(59,102)	(-43,10)	-8,36
MM_2440	(77,84)	(-3,5)	-7,85
MM_1553	(61,89)	(4,42)	-7,61
MM_2527	(119,131)	(-3,8)	-7,58
MM_1448	(124,136)	(-42,-30)	-7,54
MM_0339	(76,89)	(-14,-1)	-7,28
MM_0690	(80,88)	(-11,-3)	-7,11
MM_2321	(54,87)	(-15,23)	-7,05
MM_2320	(1,11)	(-47,-37)	-6,72
MM_1244	(119,126)	(1,8)	-6,21
MM_3184	(122,131)	(-29,-20)	-5,92
MM_2888	(58,102)	(-51,-7)	-5,89
MM_0716	(122,130)	(-23,-15)	-5,86
MM_0591	(66,74)	(-18,-10)	-5,76
MM_1139	(137,153)	(13,27)	-5,76
MM_1059	(122,134)	(-43,-27)	-5,58
MM_2005	(148,160)	(-41,-29)	-5,45
MM_1242	(119,126)	(4,11)	-4,49
MM_1543	(66,105)	(-36,3)	-4,21
MM_0842	(125,132)	(-20,-13)	-4,15
MM_1140	(130,143)	(-36,-24)	-4,00
MM_3262	(131,138)	(-46,-39)	-3,95
MM_1138	(68,76)	(-38,-30)	-3,52
MM_0804	(24,41)	(4,21)	-3,17
MM_1397	(147,156)	(-24,-15)	-2,96
MM_0542	(65,72)	(7,14)	-2,50
MM_0002	(51,58)	(20,27)	-2,19
MM_0977	(2,9)	(-17,-10)	-1,32
MM_0898	(68,75)	(-28,-21)	-1,20
MM_1545	(49,61)	(-1,11)	-1,02
MM_2230	(155,162)	(-13,-6)	-0,85
MM_0924	(54,79)	(-12,12)	-0,79
MM_0241	(77,100)	(20,48)	-0,47
MM_0021	(69,76)	(3,10)	-0,36
MM_2323	(5,12)	(2,9)	-0,30

Table S5. Predicted interactions with $sRNA_{162}$ for ORFs with increased gene expression in microarrays upon $sRNA_{162}$overexpression.

Interactions were computed with the tool IntaRNA (48). Predicted interaction site positions are given for the mRNA relative to the start codon and for $sRNA_{162}$. The interaction energy includes the interaction site unfolding energies and the duplex energy. In addition, predicted SD sequence positions and the change in their unpaired probability due to interaction formation (ΔPU SD) are given for all upregulated ORFs.

Target	Interaction site $sRNA_{162}$	Interaction site target	SD position target	ΔPU SD	Interaction energy [kcal/mol]
MM_2962	(116,134)	(-126,-107)	(-12,-6)	0,005	-10,30
MM_1688	(1,11)	(-56,-45)	(-14,-8)	0,002	-8,04
MM_2049	(121,139)	(-122,-102)	(-12,-6)	0,003	-7,38
MM_2961	(121,136)	(-102,-84)	(-33,-27)	0,004	-4,51
MM_1690	(1,11)	(-47,-35)	(-13,-7)	0,003	-4,33
MM_3334	(1,10)	(-142,-133)	(-16,-10)	0,012	-3,73
MM_2860	(26,35)	(-126,-117)	(-13,-7)	0,113	-2,65
MM_1693	(59,99)	(-182,-134)	(-12,-6)	0,018	-2,03
MM_2047	(4,12)	(-185,-177)	(-13,-7)	0,048	-1,11
